# Routine use of patient-reported experience and outcome measures for children and young people: a scoping review

**DOI:** 10.1186/s13643-024-02706-x

**Published:** 2024-11-28

**Authors:** Anne Alarilla, Katharine Terrell, Paula Kelly, Heather Chesters, Faith Gibson, Geralyn Oldham, Debbie Sell, Gwyneth Davies, Jo Wray

**Affiliations:** 1 UCL Great Ormond Street Institute of Child Health, London, UK; 2 The Health Foundation, 8 Salisbury Square, London, UK; 3 Centre for Outcomes and Experience Research in Children’s Health, Illness and Disability (ORCHID), Great Ormond Street Hospital for Children NHS Foundation Trust, London, UK; 4 Great Ormond Street Institute of Child Health Library, University College London, London, UK; 5 School of Health Sciences, University of Surrey, Guildford, Surrey UK; 6 DRIVE, Great Ormond Street Hospital for Children NHS Foundation Trust, London, UK; 7 Respiratory Medicine, Great Ormond Street Hospital for Children NHS Foundation Trust, London, UK; 8 Institute of Cardiovascular Science, University College London, London, UK

**Keywords:** Patient-reported outcome measures (PROMs), Patient-reported experience measures (PREMs), Pediatrics, Scoping review

## Abstract

**Background:**

Patient-reported outcome measures (PROMs) measure people’s views of their health status whereas patient-reported experience measures (PREMs) are questionnaires measuring perceptions of their experience whilst receiving healthcare. PROMs/PREMs have the potential to enable children and young people (CYP) to be involved in decisions about their care and improve the quality of their care but it is not clear how often PROMs/PREMs are incorporated as part of standard care of CYP in the hospital setting. The aims of this scoping review were to understand the extent of the literature and map available evidence on the use, benefits, barriers and facilitators of PROMs/PREMs as part of standard care and treatment of CYP in hospitals.

**Methods:**

The Joanna Briggs Institute review process was used to map existing evidence on the use of PROMs/PREMs in routine care of CYP in different hospital settings worldwide. Key search terms were developed and Ovid (Emcare, Embase MEDLINE, APA PsychInfo), Scopus and Web of Science were searched. Data were analysed using frequency counts and basic content analysis for thematic mapping according to the research questions. We undertook an initial search in February 2021 and updated this in April 2023.

**Results:**

The search yielded 68,004 studies, 388 were eligible for full text review and 172 met the inclusion criteria. PROMs were more commonly used than PREMs in routine care of CYP in hospitals; these were mostly collected using electronic collection and concentrated in specific specialities, settings, contexts and countries. The findings mapped the use of PROMs/PREMs, including how data are applied in clinical practice and used for service development, but this was not consistently reported. There are specific challenges in the implementation of PROMs/PREMs in routine care of CYP that need to be considered.

**Conclusion:**

PROMs/PREMs have the potential to improve care for CYP in hospital settings contributing to different aspects of care. A better understanding of their use, how results can be applied in clinical practice and contribute to service development will enable meaningful employment. The popularity of electronically collected and captured PROMS/PREMs warrants further investigation to enable their meaningful use in routine care of CYP.

**Systematic review registration:**

Not pre-registered.

**Supplementary Information:**

The online version contains supplementary material available at 10.1186/s13643-024-02706-x.

## Introduction

Children and young people (CYP) want to be heard and involved in decisions about their health and care; however, they sometimes feel that they lack the understanding and confidence to contribute or do not feel that they are allowed to [1]. Patient-reported measures, such as patient-reported outcome measures (PROMs) or patient-reported experience measures (PREMs), are standardised measures that have the potential to involve CYP directly (including those as young as 5 years old) [2] and/or their parents/carers in decisions about their care [3]. PROMs are reports of symptoms, functioning, health perceptions and/or health-related quality of life (QoL) [4, 5], whereas PREMs are reports of people’s experiences of the process of care that may cover themes including communication with staff, care received, shared decision-making and consideration of hospital or ward environment [5, 6]. In pediatric care, PROMs/PREMs can enhance the quality of care by improving communication and health-related QoL, identify unmet needs and facilitate shared decision-making [3, 7–10]. A previous review highlighted the potential benefits of integrating PROMs as part of standard care [7]; however, it did not investigate the impact after implementation. Another review explored the types of PREMs used as part of pediatric care [8]; however, this review focused on high-income countries and the use of validated measures only. Additionally, from both reviews, it is unclear whether the intended benefits of using PROMs/PREMs are realised once implemented, where they are being implemented, and whether there are differences in how PROMs and PREMSs are used as part of routine pediatric care.

The use of PROMs/PREMs in routine care does present opportunities to improve care for CYP but may also present distinct challenges that may influence the implementation of routine collection. CYP of varying ages, developmental and cognitive abilities may not reliably complete PROMs/PREMs as intended [2, 11]. Furthermore, the care of CYP typically involves families/caregivers which introduces ethical concerns in relation to privacy and consent [9]. However, the extent to which these challenges affect the routine use of PROMs/PREMs in the pediatric hospital setting is unclear. Therefore, it is also possible that there are specific barriers and facilitators to the use of PROMs/PREMs in pediatric care.

The aims of our scoping review were to understand the extent of the literature and map the available evidence on the use, benefits and barriers and facilitators of PROMs/PREMs as part of standard care and treatment of CYP in hospital. A scoping review was chosen because our research questions were explorative in nature and it allowed us to objectively summarise the breadth of the literature (including different sources) and identify knowledge gaps within this field [12].

## Review questions

1. How are PROMs/PREMs used to assess the experience and outcomes of CYP’s care and treatment in hospital?
2. How are PROMs/PREMs data applied in clinical practice?
3. How do PROMs/PREMs contribute to service development?
4. Are there any patient groups for whom PROMs/PREMs are not an integral part of routine care provision?
5. What is the evidence on the availability and utilisation of reports generated from CYP and proxies?
6. What are the barriers and facilitators to the utilisation of PROMs/PREMs in routine hospital care for CYP?

## Methods

This scoping review was conducted in accordance with the Joanna Briggs Institute (JBI) methodology for scoping reviews [13] building on Arksey and O’Malley [14] and Levac et al. [15]. The Preferred Reporting Items for Systematic Reviews and Meta-Analyses extension for Scoping Review (PRISMA-ScR) [16] checklist was used to guide the reporting. A review protocol was not published.

### Eligibility criteria

The eligibility criteria were established in relation to the research questions and using the population, concept and context framework (PCC). The full inclusion and exclusion criteria can be found in Additional File 1.

### Participants

Studies were included if the data were from CYP, or their proxies (e.g. parents, carers or guardians). CYP are defined for this purpose as from birth (including neonatal) to 25 years old [17] in children’s or adolescent and young adult (AYA) settings. Studies including clinicians working with children and young people or their proxies were also included.

### Concept

PROMs measure patients’ views of their health status; PREMs are questionnaires measuring the patients’ perceptions of their experience whilst receiving care [5]. Included studies described routine collection of PROMs/PREM data.

### Context

CYP treated by professionals based in pediatric hospital settings were included, including virtual clinics. This included AYA settings where data from those <25 years could be separated from those >25 years of age. Patients not treated in children’s or AYA settings and those whose treatment was not led by hospital-based services or who were seen in other clinical settings; e.g., primary care were excluded.

### Type of sources

Only studies in English language were included. We included descriptive observational study designs including cohort studies, case series, individual case reports and descriptive cross-sectional studies, and qualitative studies. A pragmatic decision was made to include studies published since 2008 as this marked the start of routine collection of PROMs in the UK [18]. Both published and unpublished literature (e.g. conference proceedings) were considered for inclusion.

Data primarily collected as part of a research study (e.g. randomised controlled trials), rather than routine clinical care, were excluded. Systematic reviews were also excluded. Text and opinion pieces and theses were reviewed but if they did not include explicit details about PROM/PREM use in a relevant setting, were a commentary on another article or comprised a series of published articles (e.g. a thesis), they were also excluded. For theses and opinion pieces cited, literature was considered for inclusion.

### Search strategy and information sources

An initial search was performed on 21 February 2021 and included sources published from 1 November 2008. To capture the most recent evidence at the time, an updated search was performed for the period 22 February 2021 to 4 April 2023. The databases Embase, EMcare, MEDLINE, PsycINFO, CINAHL Plus (EBSCOhost), Scopus and Web of Science were searched. The final search terms including an example of a search strategy are included in Additional File 2.

### Study selection and charting the data

Following the searches, all identified citations were collated and uploaded into Covidence (Veritas Health Innovation, Melbourne, Australia) and duplicates removed. Titles and abstracts were screened independently by two reviewers for assessment against the inclusion criteria for the review. Potentially relevant sources were retrieved in full. The full text of selected citations was assessed in detail against the inclusion criteria by two or more independent reviewers. Reasons for exclusion of sources of evidence at full text that did not meet the inclusion criteria were recorded. Disagreements between reviewers were resolved through discussion, or with an additional reviewer/s.

### Data extraction

Data were extracted from the selected sources by one reviewer using a data extraction tool developed by the review team, and a minimum of 10% was reviewed by a second reviewer. The extraction form (Additional File 3) is based on Peters et al. [13].

### Data analysis

Data were analysed using frequency counts for different demographic characteristics of included studies and basic qualitative content analysis in relation to the research questions. An inductive approach was taken as recommended by Pollock et al. [19]. Frequency counts were undertaken in R (code available on GitHub) or Excel. Basic qualitative content analysis was undertaken in Mural, with categorization available in Mural.

## Results

**Fig. 1:**
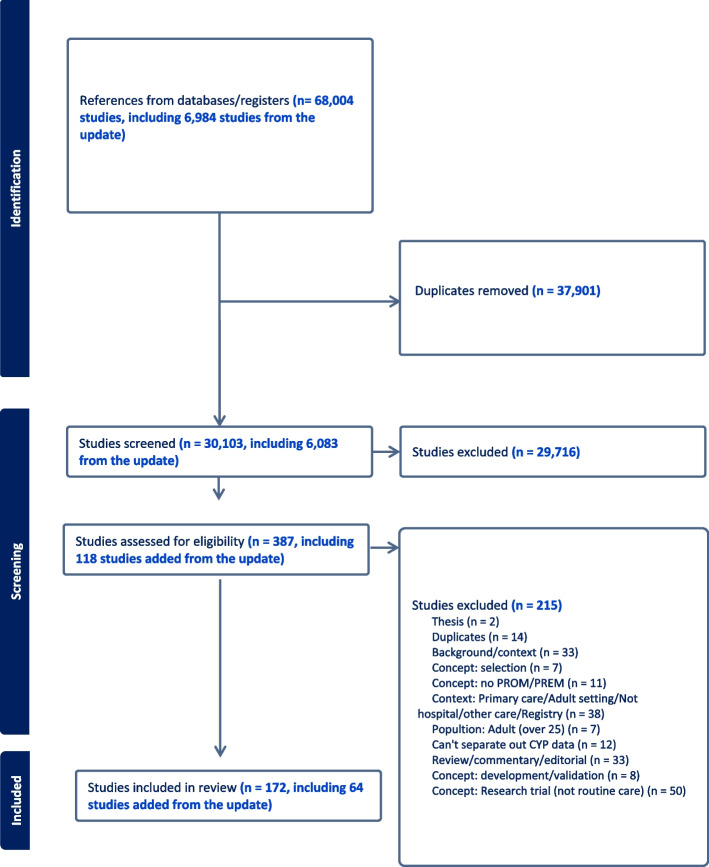
PRISMA ScR flow diagram

The PRISMA ScR flow diagram summarises the process of study selection (Fig. 1). The search identified 68,004 references, and after the removal of duplicate titles and abstracts (*n*=37,901), 30,103 sources were screened. A total of 387 were eligible for full text review and 172 met criteria for inclusion in the final review. A total of 147 (85%) sources included data on PROM use only [20–165]; 11 (6%) included data on PREM use only [166–176] and 14 (8%) included data on both PROM and PREM use [9, 177–189]. Details about the study (country, type of data collection, inpatient/outpatient setting and participants) are provided in Table 1. A detailed summary of details of the included studies can be found in Additional File 4, and full characteristics including measures used can be found in Table 2.

**Table 1:** Summary characteristics of included studies

**Characteristics**	***N*** ** = 172** (*N*, %)
**Collection type**	
Electronic	79 (46%)
Mixed	8 (5%)
Pen and paper	14 (8%)
Telephone	2 (1%)
Not applicable	13 (8%)
Not stated	56 (33%)
**Type**	
PROMs	147 (85%)
PREMs	11 (6%)
PROMS and PREM	14 (8%)
**Participant**	
Patient	51 (30%)
Proxy	12 (7%)
Patient and proxy	45 (26%)
Clinician	23 (13%)
Clinician and proxy	10 (6%)
Patient, proxy and clinician	17 (10%)
Not stated/not clear	11 (6%)
**Setting**	
Outpatient	73 (42%)
Inpatient	13 (8%)
Mixture	6 (3%)
Not stated	80 (47%)
**Specialties**^a^	
Oncology	17 (10%)
Endocrinology	12 (7%)
Mental health	11 (6%)
Multiple^b^	11 (6%)
Not stated	26 (15%)
Other specialties	95 (55%)
**Context**	
Multicentre	34 (20%)
Single centre	120 (70%)
Registry network	5 (3%)
Not stated	13 (8%)
**Country**^b,c^	
United States	53 (31%)
Netherlands	39 (23%)
United Kingdom	26 (15%)
Canada	14 (8%)
Other	40 (23%)
Not stated	6 (3%)

^a^Individual specialties reported only include studies with one speciality, ‘Multiple’ are studies reporting multiple specialties and ‘Not stated’ are studies that do not specify any specific specialties

^b^Some studies reported the individual specialties included but others mentioned working across departments, centres or hospitals and did not indicate the specific specialties

^c^Other’ countries are reported individually in the Additional File 4 which encompasses all countries including those of lower and middle income, yet these were noted to be underrepresented

**Table 2:** Characteristics of all include studies

Author	Type	Country	Collection	Context	Participant Details	PROMs	PREMs	Speciality
Ng 2020 [99]	PROMs	Multiple countries	Pen and paper	Registry network	Liver transplant recipients aged 8-18years old >1-year post first isolated pediatric liver transplant	Pediatric liver transplant quality of life (PeLTQL)		Transplant
Naranjo 2017 [98]	PROMs	United States	Not stated	Single centre	113 families All pts aged ≥ 12 years (*n*=54) All caregivers also screened if caregivers of patients <12 screened positively for depression or anxiety, the patients were also screened (ages 8-11) or interviewed (ages 6-8)	PHQ-9 GAD-7		Respiratory
Mims 2019 [183]	PROMs and PREMs	United States	Not stated	Single centre	256 patients	3 pro questions, focusing on health, QOL and treatment burden	1 question on satisfaction with team interactions	Respiratory
Murphy 2017 [96]	PROMs	United Kingdom	Not stated	Multicentre	17 patients with erythropoietic protoporphyria (aged unknown)	Erythropoietic protoporphyria quality of life (EPP-QOL)		Dermatology
Mentrikoski 2018 [88]	PROMs	United States	Electronic	Single centre	All patients (and their caregivers) who received treatment through the inpatient and outpatient burn centres.	Quality of life (specific tool not stated)		Burn
Maxwell 2015 [87]	PROMs	United States	Not stated	Single centre	20 young people aged over 10	PedsQL sickle cell disease module (PedsQL SCD)		Haematology
Mager 2019 [84]	PROMs	Canada	Not stated	Single centre	CYP (and parents) aged 8-17y (18m/17f) who underwent liver transplantation	Pediatric liver transplant quality of life questionnaire (PeLTQL) parent proxy and child reports		Transplant
Limperg 2013 [80]	PROMs	Netherlands	Electronic	Single centre	106 children and their parents	TNO-AZL Preschool children Quality of Life (TAPQoL) - Pediatric Quality of Life Inventory (PedsQL) - Strength and Difficulties Questionnaire (SQD)		Haematology
Limperg 2017 [81]	PROMs	Netherlands	Electronic	Single centre		TNO-AZL Preschool Children Quality of Life (TapQoL) - Pediatric Quality of Life Questionnaire (PedsQL) - Hemophilia Quality of Life (Haemo-QoL) - Hemophilia Self-Efficacy Scale (HSES) - Strengths and Difficulties Questionnaire (SDQ) - Pediatric Hemophilia Activities List (Ped-HAL)		Haematology
Limperg 2012 [79]	PROMs	Netherlands	Electronic	Single centre	40 children under 18 with congenital bleeding disorders	TNO-AZL Preschool children Quality of Life, TAPQOL or Pediatric QoL inventory; PedsQL); SDQ		Haematology
Lewer 2011 [78]	PROMs	Australia	Not stated	Single centre	31 CYP aged 2-17years with atopic dermatitis	Edsel		Immunology
L'estrange-snowden 2018 [170]	PREMs	United Kingdom	Pen and paper	Multicentre	18,687 patients aged 0-15 who received care between Oct and Dec 2016		Children and young people’s inpatient and day case survey	Multiple specialties
Kuijlaars 2019 [75]	PROMs	Netherlands	Pen and paper	Not stated	73 boys aged 5.4-18.0 years with moderate-severe haemophilia and their parents	Pediatric hemophilia activities list (pedHAL) (53 items, 7 domains)		Haematology
Kroupina 2020 [73]	PROMs	United States	Not stated	Single centre	10 patients under 1 year old and their parents	Ages & Stages Questionnaires: Social-Emotional (ASQ:SE) (2nd ed)		Mental health
Knottnerus 2017 [72]	PROMs	Netherlands	Electronic	Single centre	15 patients	Basic lto PedsQL PedsQL fatigue		Endocrinology
Schepers 2017 [126]	PROMs	Netherlands	Electronic	Single centre	205 children/proxies aged 0-18years and 28 clinicians	Generic HRQOL PROMs PedsQL 3.0 acute cancer module		Oncology
Schepers 2016 [123]	PROMs	Multiple countries	Not applicable	Single centre	352 health care professionals (hcps) (43% male) -pediatric oncologist/hematologist, nurse practitioner, pediatric (neuro)surgeon, pediatrician, pediatric neurologist	None reported		Oncology
Schepers 2014 [122]	PROMs	Netherlands	Electronic	Multicentre	74 parents of children; 21 pediatric oncologists	TAPQOL PedsQL 4.0		Oncology
Kermarrec 2015 [70]	PROMs	United Kingdom	Not stated	Single centre		Ages and stages questionnaire (ASQ)		Occupational therapy
Katsicas 2011 [182]	PROMs and PREMs	Argenti	Not stated	Single centre	12 patients, age at onset 96 (9-18 months)	Outcome measures designed and applied	A proposed set of quality measures for the process of care in JSS was designed and applied (unclear if PREM)	Rheumatology
Santana 2015 [118]	PROMs	Netherlands	Not applicable	Single centre	Training directed at pediatricians utilizing KLIK. no details provided of numbers participating in training patients (children) and /or parents completed questionnaires at home via website	PedsQL KLIK		Multiple specialties
Salmond 2020 [117]	PROMs	United Kingdom	Not stated	Single centre	67 young people aged 12-18years old	Revised child anxiety and depression scale (RCADS) Young person’s clinical outcomes in routine evaluation (YP CORE) Moods and feelings questionnaire (MFQ)		Mental health
Haverman 2013 [63]	PROMs and PREMs	Netherlands	Electronic	Multicentre	176 children aged 0-18years with juvenile idiopathic arthritis visiting clinic between Feb 2009-Feb 2010	Netherlands Organisation for Applied Scientific Research-Academisch Ziekenhuis Leiden (TNO-AZL) Preschool Children Quality of Life (TAPQOL) PedsQL Generic Core Scale (Generic HRQoL scale) Self-composed measure based on DISABKIDS arthritis module Childhood Health Assessment Questionnaire (CHAQ) Visual Analogue Scale (VAS) (pain and overall well-being)	Adapted patient satisfaction questionnaire on vas, covering: (1) meeting needs, (2) active involvement, (3) provided information, (4) (emotional) support, and (5) overall satisfaction with care provided during the consultation	Rheumatology
Haverman 2017 [181]	PROMs and PREMs	Netherlands	Electronic	Multicentre	-Over 1100 patients in 13 patient groups; 113 professionals from 5 centres (2014) -Over 1450 patients in 17 patient groups; 160 professionals (2014); -Over 6500 patients in 61 patient groups; 500 professionals from 11 hospitals (2017)	As of Aug 2013: HRQOL Generic: - Pediatric Quality of Life Inventory (PedsQL) PedsQL Fatigue module TNO-AZL Preschool children Quality of Life (TAPQOL) HRQOL Disease-specific: PedsQL Transplant module: nephrology PedsQL Cancer module: oncology Childhood Health Assessment Questionnaire (CHAQ): rheumatology Dutch Defecation Questionnaire (DDL): gastroenterology Questionnaire Juvenile Idiopathic Arthritis Medication and appearance after kidney transplant Medication and transition questions HIV MIND Youth Questionnaire (MY-Q): diabetes Questionnaire Cleft lip - Satisfaction with Appearance (SwA): cleft lip - Questionnaire Spherocytosis Psychosocial screening tools: Strengths and Difficulties Questionnaires (SDQ) Children’s Revised Impact of Event Scale (CRIES) Child Behavior Checklist (CBCL) Behavior Rating Inventory of Executive Function (BRIEF) Questionnaire for Behavioral Problems in Children (VvGK) Other: Course of Life questionnaire (LVJV/CoL) Questions about school	Evaluation of KLIK ePROfile	Multiple specialties
Haverman 2013 [180]	PROMs	Netherlands	Electronic	Single centre	20 families screened - 19 mothers, 16 fathers, 4 children (all 8years or above)	Hrqol, distress, ptsd		Multiple specialties
Haverman 2015 [64]	PROMs	Netherlands	Electronic	Single centre	Young adults	Not stated		Transition/Gender Services
Hacker 2017 [59]	PROMs	United States	Pen and paper	Single centre		PHQ-9		Respiratory
Gmuca 2019 [54]	PROMs	United States	Electronic	Single centre	65 pts aged 8-17 years with juvenile fibromyalgia syndrome (jfms) and their proxies	Pediatric quality of life short form 15 generic core scales (PedsQL SF-15		Multiple specialties
Devereux 2009 [37]	PROMs	United Kingdom	Not stated	Single centre		Children’s dermatology life quality index (CDLQI)		Dermatology
Riobueno-ylor 2019 [107]	PROMs	United States	Electronic	Multicentre	Parents of 147 children 5-18years old; clinicians in outpatient setting (physicians, nurse practitioner, registered nurse), 79 clinicians in surgical setting (physicians 100%)	Parent reported - burn outcomes questionnaire for children ages 5-18 years (BOQ 5-18) Pediatric symptom checklist (PSC-17)		Burn
Einaudi 2013 [44]	PROMs	France	Not stated	Single centre	78 clinicians: 19 obstetricians, 45 neontologists and 14 pediatric neurologists	Not stated		Multiple specialties
Eilander 2016 [43]	PROMs	Netherlands	Electronic	Multicentre	10 diabetes teams (26 members incl. diabetes nurses, pediatricians, psychologists and dieticians) were interviewed - 36 team members, 29 adolescents and 66 parents surveyed	Mind-youth (monitoring individual needs in young people with diabetes) (DM-Y) questionnaire (MY-Q)		Endocrinology
De Wit 2010 [38 ]	PROMs	Netherlands	Not stated	Single centre		Dawn mind youth		Endocrinology
Davis-dao 2020 [167]	PREMs	United States	Mixed	Single centre	Children receiving urological care between 2017-2019, median age 5y, 78% male		National research corporation health patient survey in English and Spanish.	Urology
Dharmaraj 2019 [40]	PROMs	Canada	Not stated	Single centre	86 post-liver transplant patients > 8 years old (median range 10-12 years old)	Pediatric liver transplant quality of life (PeltQL)		Transplant
Cunningham 2018 [34]	PROMs	United States	Pen and paper	Single centre	Patients aged 8-18 years old with functional abdominal pain disorder -Survey completed by 18 gastroenterology providers who routinely see CYP with functional abdominal pain disorders	Screen for child anxiety and related disorders (scared)-child report Functional disability inventory (FDI)-child version Numeric rating scale for pain levels		Gastroenterology
Cassidy 2017 [30]	PROMs	United States	Not stated	Single centre	17 patients ≥12 years old participating in exercise program and have completed Mental Health Screening	Patient health questionire-9 (PHQ-9) Generalized anxiety disorder-7 (GAD-7) Cystic fibrosis questionnaire-revised (CFQ-R) Habitual activity estimation scale (HAES)		Mental health
Carberry 2016 [29]	PROMs	United States	Electronic	Single centre		Not stated		Not stated speciality
Bower 2020 [26]	PROMs	United States	Pen and ppr	Single centre	55 patients over 3-month study period; 49 completed prom (89%)	Memorial symptom assessment scale		Palliative
Caddell 2015 [166]	PREMs	Canada	Not stated	Single centre	Parents of all new outpatients in June, July, August 2012 were asked for satisfaction data - 85% (n=74) responded	Adapted from Margaritis et al. exceeding parent’s expectations in ear, nose and throat outpatient facilities: the development and analysis of a questionnaire. Evaluation program planning. 24 item questionnaire, Likert scoring (strongly disagree-strongly disagree + category)		Cardiology
Burden-teh 2013 [28]	PROMs	United Kingdom	Not stated	Multicentre	Consultants (one representative per centre). Of 79 respondents, 56 filled in questionnaire	Dermatology life quality index (DLQI)		Not stated speciality
Blaauboer 2017 [25]	PROMs	Netherlands	Not applicable	Single centre	54 children (all children with severe skin disease at the setting), mean age 9.5 years, sd = 5.2, 42.6% female (4-18 years)	Pediatric quality of life inventory for children (PedsQL 4.0, 6-18 years)		Dermatology
Racine 2018 [104]	PROMs	Canada	Not stated	Single centre	16-year-old female with cancer	Pediatric quality of life inventory (PedsQL) 4.0		Not stated speciality
Anthony 2021 [22]	PROMs	Canada	Electronic	Multicentre	63 participants: 20 patients, 22 caregivers, 21 hcps (all English speaking with no severe cognitive impairment)	Participants reviewed PedsQL generic core scales and PedsQL transplant module. The eProm platform was also reviewed		Transplant
Roesler 2018 [112]	PROMs	United States	Not stated	Single centre	Pts aged 6-18y with pediatric illness associated with psychiatric comorbidity, and their families	Family assessment device		Not stated speciality
Murphy 2011 [95]	PROMs	United States	Electronic	Single centre	Parents of 183 children aged under 18y (105m, 78f)	Pediatric symptom checklist (PSC) (parent-report) - 35 items rated from 0 (never) to 2 (often)		Psychiatry
Wray 2020 [176]	PREMs	United Kingdom	Not applicable	Single centre	565/1876 parents of children receiving cardiothoracic care		14 different PREMs developed for each ward/service	Not stated speciality
Stevens 2012 [135]	PROMs	Canada	Not stated	Multicentre	3,822 children aged 0 to 18 years hospitalized for more than 24 hours	28% of pain assessments used measures validated for that age group; 5.4% of pain assessments use validated pain measure that was age inappropriate		Not stated speciality
Marker 2019 [86]	PROMs	United States	Electronic	Multicentre	Settings 4 clinics at 4 hospital sites & outreach clinics	Patient Heath Questionnaire (phq-2, phq-4, phq-9)		Endocrinology
Maasalo 2017 [83]	PROMs	Finland	Not stated	Single centre	62 psychiatric patients aged 5-12years	Parent-rated strengths and difficulties questionnaire (SDQ) (25 item instrument measuring emotional and behavioral problems)		Psychiatry
Edbrooke-childs 2016 [42]	PROMs	United Kingdom	Not applicable	Single centre	28 clinicians who attended 1-day training (25f, 3m) incl. psychotherapists (5), clinical leads (3), trainee psychotherapists (3), mental health workers (2)	Not stated		Mental health
Brann 2018 [27]	PROMs	Australia	Pen and ppr	Single centre	532 adolescents (12-17years, mean 15.3years, sd=1.3) and 125 young adults (18-25years, mean 19.1years, sd=1.6) seen for an initial outpatient appointment in a mental health service between July 2012 and June 2014. 71% female	Young adult strengths and difficulties questionnaire (SDQ)		Mental health
Sharma 2020 [130]	PROMs	Multiple countries	Not applicable	Multicentre	109 allergists	None specified		Allergy
Hall 2014 [60]	PROMs	United Kingdom	Not stated	Multicentre	10 clinicians (5 clinical psychologists, 2 mental health nurses, 1 nurse prescriber, 1 consultant psychiatrist, 1 trainee psychiatrist; 8f, 2m); 8 admin staff; 15 families - young people aged 11-19y (m=5=15years, sd1.9; 8f, 7m)	Strengths and difficulties questionnaire (SDQ)		Mental health
Haverman 2019 [65]	PROMs	Not stated	Electronic	Not stated		Around 300 proms available on KLIK website in the following categories: Generic HRQOL [eg, pediatric quality of life inventory (PedsQL)] Disease-specific HRQOL (eg, PedsQL transplant module) Daily functioning [eg, child health assessment questionnaire (CHAQ)] Cognitive functioning [eg, behavior rating inventory of executive functioning (BRIEF)] Symptoms [eg, pediatric ulcerative colitis activity index (PUCAI)] Psychological screening [eg, strengths and difficulties questionnaire (SDQ), hospital anxiety and depression scale (HADS)] Transition [eg, skills for growing up nephrology (SGU-N) tool]		Not stated speciality
Aldekhyyel 2018 [21]	PROMs	United States	Electronic	Single centre	2,447 CYP who had at least one pain medication administered in 22-month period (11 months before and 11 months after implementation of new system) of the patients/parents who used the system: 45% aged 10-18 54% male 75% white	27,224 times the PMI triggered pain assessment. 6.5% of these (1767 times) were responded to by 608 unique patient/parents		Pain
Griffiths 2017 [56]	PROMs	United Kingdom	Not stated	Single centre	161 young people who had face-to-face contacts with clinicians in 2-year period at three services	World health organization quality of life-brief (WHOQOL-BREF) Subscales from beck youth inventories Positive and negative syndrome scale Beck depression inventory-ii		Multiple speciality
Gerhardt 2018 [53]	PROMs	United States	Electronic	Single centre		Not stated		Not stated speciality
Fullerton 2018 [52]	PROMs	United Kingdom	Not stated	Single centre	41 clinical supervisors (31f, 10m) - psychologists (22%), family therapists (12%), counsellors (10%), child and adolescent psychotherapists (7%), other unqualified staff (7%), occupational therapists (5%), medics (5%), nurses (5%), primary mental health workers (5%), creative therapists (2%), educational professionals (2%), other qualified staff (2%), unspecified (16%)	None reported		Mental health
Engelen 2012 [46]	PROMs	Netherlands	Electronic	Single centre	Children with cancer (0-18 years) shortly (0-3 months) after completion of treatment (stem cell transplantation), their parents and treating pediatric oncologists	PedsQL generic core scale (pediatric quality of life inventory) TapQOL (TNO-AZL preschool children quality of life)		Oncology
Engelen 2010 [45]	PROMs	Netherlands	Electronic	Single centre	Pediatric oncologists	PedsQL Tapqol		Oncology
Engelen 2012 [47]	PROMs	Netherlands	Not stated	Multicentre	94 patients (intervention); 99 patients (control); 34 oncologists (47.1% female)	Pediatric quality of life inventory (PedsQL) generic core scale TNO-AZL preschool children quality of life (TapQOL)		Oncology
Edbrooke‐Childs 2017 [41]	PROMs	United Kingdom	Not applicable	Multicentre	109 clinicians, 85% female, median age 53-44years	Not stated		Mental health
Cox 2021 [33]	PROMs	United States	Not applicable	Single centre	Health system leaders (56%), including directors of clinical or academic programs and initiatives Measurement implementers (72%), and ambulatory pediatric clinicians (39%) generalists or subspecialists in areas like endocrinology, neuropsychology, or pediatric surgery most employed in academic medical institutions (83%)	Not stated		Not stated speciality
Perito 2021 [102]	PROMs	United States	Electronic	Registry network	114 parent-child dyads	Pediatric liver transplant quality of life (PeltQL)		Transplant
Kemp 2018 [169]	PREMs	Canada	Telephone	Multicentre	3389 parents/guardians of patients under 18years old, (55.1%m, 44.9%f)		Modified child hospital consumer assessment of healthcare providers and systems (child HCAHPS)	Mental health
Barthel 2016 [23]	PROMs	Germany	Electronic	Multicentre	312 children and adolescents (mean age 12.5years, sd=2.8, 47.1% female) with asthma (18.5%), diabetes (65.9%) or rheumatoid arthritis (15.6%), and 8 pediatricians (mean age 43.4years, range 38-52), 4 from each clinic, 50% female, with sub-specialties in diabetology, pulmonology or rheumatology	Kids-cat - first German cat measuring HRQOL in children and adolescents aged 7-17 years. The kids-cat tool covers the five dimensions of physical well-being, psychological well-being, parent relations, social support and peers, and school well-being, which follow the domain structure of kidscreen-27		Multiple speciality
Wolfe 2014 [188]	PROMs and PREMs	United States	Electronic	Multicentre	104 patients and their parent/s and clinicians	PQ memorial symptom assessment scale (MSAS) Pediatric quality of life inventory 4.0 generic core scales (PedsQL 4.0) Sickness question developed asking how sick the child felt in the last week, between "not sick at all" and "very sick"	Child and parent surveys assessing satisfaction with the PQ intervention, adapted from existing questionnaires	Oncology
Teela 2020 [142]	PROMs	Netherlands	Electronic	Single centre	104 patients with chronic kidney disease	Pediatric quality of life inventory for children (PedsQL) TNO-AZL preschool children quality of life (TapQOL)		Nephrology
Swales 2016 [136]	PROMs	United Kingdom	Pen and ppr	Single centre	43 young people in dialectical behavior therapy (dbt), aged 14-18y with 5 or more bpd criteria including recent self-harm	Euroqol 5 dimensions (EQ-5D)		Psychology
Aberdeen 2019 [20]	PROMs	United States	Not applicable	Single centre	72 members of pediatric research in sports medicine society (prism) - Orthopaedic surgeons (56; 78%), sports medicine primary care physicians (14; 19%), nurse practitioners/physician assistants (3; 4%)	Neck-specific PROs Back-specific PROs Shoulder-specific pros (Dash, Quickdash and other "other" (incl. activity and fear avoidance scales)) Hip-specific PROs Knee-specific PROs (IKDC, Pedi-IKDC, LEFS, knee outcome survey, KOOs, KOOs-Child, Other) Ankle pros Additional PROs Affective domain/QOL scales (Pedi-Fabs, ACL-RSI, PROMIS)		Orthopaedic
Huang 2012 [68]	PROMs	United States	Not stated	Single centre	453 physicians of general pediatrics (*n*=182) and 7 selected pediatric subspecialties (*n*=271)	Not stated		Not stated speciality
Hames 2016 [61]	PROMs	United Kingdom	Electronic	Single centre	187 young people aged 15-23y (mean 18.0y), 52.9% female	Imparts core set includes: Patient Health Questionnaire (PHQ9) Generalized Anxiety questionnaire (GAD7) The Liver Transition Battery additionally includes: Brief Illness Perception questionnaire (BIPQ) A modified distress thermometer (DT)		Transition
Bjertnaes 2018[ 177]	PROMs and PREMs	Norway	Pen and ppr	Registry network	Parents of 2606 patients registered in the Norwegian childhood diabetes registry, aged 0-17, with type 1 diabetes, with 1 outpatient consultation in previous year	No PROM used, however the PREM includes 5 items relating to outcomes	Developed for this trial. Includes organization, consultation, equipment, nurse contact, doctor contact and outcome.	Endocrinology
Zia 2016 [165]	PROMs	United States	Pen and ppr	Single centre	Adolescent girls aged up to 21years old	PedsQL		Haematology
Bele 2022 [190]	PROMs	Canada	Electronic	Multicentre	17 clinicians and administrators from the Alberta children's hospital (ACH) outpatient asthma and community clinics. 13 working at ACH outpatient asthma clinics	Pediatric quality of life inventory (PedsQL) version 4.0 generic core scales and PedsQL asthma specific module		Respiratory
Cheng 2022 [32]	PROMs	United States	Electronic	Multicentre	40,603 adults and 19,289 children	Patient-reported outcomes measurement information system (PROMIS)- pediatric mobility version 1.0 or 2.0, upper extremity version 1.0, pain inference version 1.0 or version 2.0, pediatric peer relationships version 1.0		Orthopaedic
Chua 2023 [178]	PROMs and PREMs	Singapore	Electronic	Single centre	451 patients with adolescent idiopathic scoliosis and 279 underwent bracing. mean age 14.6 years in observation group, mean age also 14.6 years in bracing group	Scoliosis research society-22 revised (SRS-22r) European quality of life five dimension five-level version (EQ-5d-5l) EQ-visual analogue scale scores	Using own institution outpatient experience feedback form adapted from the hospital consumer assessment of healthcare providers and system survey	Not stated speciality
Dalton 2022 [35]	PROMs	United Kingdom	Not stated	Single centre	519 parents and 248 children (age 7-16) with single suture craniosynostosis	Surgical outcome questions- asked about how noticeable the child's head shape is and how much this bothers the parent and/or child		Neurology
Delgiudice 2022 [179]	PROMs and PREMs	Netherlands	Not stated	Single centre	239 patient questionnaires and 238 parents' questionnaires from 440 patients with jia diagnosed according to the ILAR criteria and enrolled in pharmachild	Juvenile arthritis multidimensional assessment report (jamar) Satisfaction with disease outcome (ja-cass or ja-pass) Available as parent proxy report of child self-report (suggested age range of 7-18 years)		Rheumatology
Dhar 2021 [39]	PROMs	India	Not stated	Multicentre	25 patients above 11 years who have received Dupilmab treatment	Efficacy was assessed by comparing scorad ('scoring atopic dematitis') and EASI (eczema area and severity index). The dermatology life quality index (DLQI)		Dermatology
Wheatbutt 2014 [161]	PROMs	United States	Not stated	Single centre	40 caregivers completed parent-reports, 25 youth and 51 adults completed self-reports	Strengths and difficulties questionnaire Revised children’s anxiety and depression scale Patient health questionnaire (PHQ9) and GAD-7		Respiratory
Fernandez-Quintana 2021 [48]	PROMs	Sweden	Not stated	Multicentre	111 psychiatric outpatients	ADHD self-report scale for adolescents (ASRS-A) ASRS-A-P for parent		Mental health
Fischer 2020 [49]	PROMs	Not stated	Electronic	Multicentre	203 patients part of the diabetic sub sample (from a clinical sample of 248 patients aged 7-17 years)	Kids-CAT		Not stated speciality
Fischmeister 2021 [50]	PROMs	Austria	Electronic	Single centre	98 children with cancer and 124 corresponding parents completed assessment before and after rehabilitation	PedsQL (children older than 5 years completed self-report version and parents provided proxy-responses)		Rehabilitation
Franklin 2021 [51]	PROMs	United States	Electronic	Single centre	24 patient-parent dyads, patients are athletes with sports injuries	Patient reported outcome measurement information system (PROMIS)		Orthopaedic
Graham 2023 [55]	PROMs	Canada	Electronic	Single centre	35 participants including 2 thalassemia patients, 4 thalassemia caregivers, 2 hemophilia patients, 6 hemophilia caregivers, 3 Sickle Cell Disease patients, 4 Sickle Cell Disease caregivers, 3 immune thrombocytopenia patients, 5 immune thrombocytopenia caregivers, and 6 Health Care Professionals	TranQOL for patients with thalassemia PEDSQL Hemophilia quality of life questionnaire (Haemo-QOL) Canadian hemophilia outcomes-kids' life assessment tool (CHO-KLAT) PedsQL SCD module Kids ITP tool (KIT)		Haematology
Gupta 2023 [58]	PROMs	Canada	Mixed	Registry network	5435 adolescent and young adults (15-29 years) with cancer	Edmonton symptom assessment system (ESAS)		Oncology
Hanmer 2021 [62]	PROMs	United States	Electronic	Single centre	45 practices participated	Routine questionnaires considered during well children care (WCC) visits Modified checklist for autism in toddlers (M-CHAT; 18 and 30 months) The patient health questionnaire (PHQ-2) for ages 11+ to screen for depression. The PHQ-9 was administered if PHQ-2 was positive The screening to brief intervention (S2BI) for ages 11+ to screen about alcohol, tobacco and marijuana. If 3 item S2BI was positive, it continued to the 12 item For children with asthma, an age appropriate asthma control test (act)		Not stated speciality
Henning 2022 [66]	PROMs	Europe	Not applicable	Single centre	Clinicians who are part of the European reference network for rare and complex epilepsies (EPICARE) - 4 psychologists, 24 physicians. 11 worked with children and nine with adults and eight with both children and adults	QOL- Quality of Life in Epilepsy 10 (qolie-10) (*n* = 9), QOLIE 31 (*n* = 9), and QOLIE 89 (*n* =5) Psychiatric comorbidity- beck depression inventory (*n* = 15), beck anxiety inventory (n = 10), hospital anxiety and depression scale (HADs) (*n* = 9), neurological disorders depression inventory for epilepsy (NDDI-E) (n = 5), general anxiety disorder-7 (GAD-7) (*n* = 5), and aberrant behaviour checklist (ABC) (*n* = 5) Wechsler scale of intelligence (WPPSI IV, WISC IV, WAIS IV), Mini-mental status examination MMSE (*n* = 8), Epitrack (*n* = 7), Boston Ming test (*n* = 6), Montreal cognitive assessment MoCA (*n* = 6), EPItrack (*n* = 5), and behaviour rating inventory of executive function brief (*n* = 5) for cognitive dysfunction Adverse events profile (AEP), Liverpool adverse events profile (ILAEP) and complaints assessment scale (CAS) were used to screen for adverse effects of anti-seizure medications (ASM) Arizona sexual experiences scale (ASEX) to measure sexual dsyfunction		Neurology
Hjollund 2023 [67]	PROMs	Denmark	Electronic	Single centre	349 children (mean age 10.7, 36.1% female)	For children-> pain NOS (proxy report)		Pain
Wang 2018 [160]	PROMs	United States	Electronic	Not stated	Parents of children with a burn injury involving a 5% of the total body surface area (TBSA) or a critical area (face, hands, feet, genitalia)	Burn outcomes Questionnaire (BOQ) for children aged 5-18 plus pediatric symptom checklist-17 (BOQ-17) (BOQ+p)		Burn
Holzman 2021 [168]	PREMs	United States	Electronic	Multicentre	Not stated		NRC health patient survey (completed by parents or guardians)	Urology
Jalilova 2023 [69]	PROMs	Turkey	Not stated	Not stated	41 youth started HCL for diabetes care completed 6th month follow up survey	Pediatric quality of life inventory (PedsQL) diabetes module 3.0 child and parents (8-12 years) Quality of life for youth (13-18 years) Strengths and Difficulties Questionnaire (SDQ) Hypoglycemia fear survey (HFS) for children The revised child and anxiety and depression scale (R-CADS) A-HCLS specific expectation and satisfaction survey		Endocrinology
Kliems 2020 [71]	PROMs	United States	Not stated	Multicentre	20 healthcare providers (physicians, nurse practitioners, social workers, and health psychologists) who care for children with asthma, type 1 diabetes, and sickle cell disease	The patient-reported outcomes measurement information system (PROMIS) family relationships short form		Not stated speciality
Kuhn 2022 [74]	PROMs	United States	Electronic	Not stated	620 patients, 90 received the electronic version	Peess v2.0-disease		Gastroenterology
Pasulo 2023 [100]	PROMs	Not stated	Electronic	Single centre	167 patients in the transition phase to adulthood	Psychological and QOL testing		Transplant
Yao 2019 [163]	PROMs	United States	Electronic	Single centre	178 parents of children (mean age 5.5years old)	Pediatric quality of life inventory (PedsQL) 3.2 diabetes module		Endocrinology
Provini 2021 [103]	PROMs	United States	Pen and ppr	Single centre	293 patients aged 13-18 years	Cutaneous body image (CBI)		Dermatology
Pryde 2021 [173]	PREMs	United Kingdom	Telephone	Single centre	24/45 families responded		Questionnaire looked at satisfaction, advantages/disadvantages over face to face and opinions on continuation of virtual appointments	Neurology
Riedl 2022 [106]	PROMs	Austria	Electronic	Single centre	236 children and 478 parents who are cancer survivors, mean age 11 (age range 5-22) and 139 are male	Pediatric quality of life inventory (PedsQL) 4.0 generic core scales PedsQL 3.0 cancer Children between 5-21 years completed the self-report version and parents independently completed a proxy version		Rehabilitation
Ross 2021 [114]	PROMs	United States	Electronic	Single centre	20 parents of children with asthma aged 5-11 years, 19 portal users, and 1 portal nonuser 17% Asian, 23% African American, 58% Caucasian, and 11% Native American (including mixed races) and 41% were Hispanic/Latino ethnicity	Not stated		Respiratory
Lassen 2023 [76]	PROMs	Denmark	Electronic	Single centre	20 children with t1d aged 11-18 years; 9 male, 11 female; 18 t1d, 2 secondary diabetes.	Diabetes eating problem survey (deps-r) The generic WHO-5 well-being		Endocrinology
Leahy 2021 [77]	PROMs	Not stated	Electronic	Not stated	7-18 year olds pediatric patients	pediatric patient-reported outcomes version of the common terminology criteria for adverse events (ped-pro-ctcae)		Transplant
Wray 2019 [175]	PREMs	United Kingdom	Not applicable	Single centre	1876 parents were asked, 30% responded		Questionnaires for parents developed for services individually, including service/condition-specific questions	Cardiology
Luijten 2020 [82]	PROMs	Netherlands	Electronic	Not stated		12 PROMIS pediatric banks translated		Not stated speciality
Wray 2017 [189]	PROMs and PREMs	United Kingdom	Not stated	Single centre	Parents of 98 infants (96% response) completed prem; PedsQL data for 64 infants (69%)	Infant PedsQL (Generic QoL measure)	Not stated	Surgery
Mandell 2021 [85]	PROMs	United States	Not stated	Single centre	164 pediatric burn patients, 81 females, 83 males mean age at injury is 6.9 years (median 6.4)	Patient reported outcomes measurement information system (PROMIS) peer relationship		Burn
Woodward 2020 [162]	PROMs	United States	Not applicable	Single centre	Clinicians/leaders (9 in focus group, 2 interviewed)	Not stated		Psychology
Mccabe 2023 [9]	PROMs and PREMs	Canada	Not stated	Multicentre	23 interviews from study 1 and 2; 2 allied health professionals, 5 researchers, 10 clinician scientists (all physicians), 2 evaluation specialists and 4 Alberta health services administrators	Not specified	Not specified	Rehabilitation
Meryk 2021 [89]	PROMs	Austria	Electronic	Single centre	12 patients aged 5-18 years age (median 7.2 years) who were treated with chemotherapy and 25% were female	ePROtect monitors patients' symptom burden during and after treatment		Oncology
Meryk 2022 [90]	PROMs	Austria	Electronic	Single centre	10-year-old male patient diagnosed with Burkitt leukemia	6-8 questions adapted from the pediatric quality of life inventory (PedsQL)		Oncology
Meryk 2022 [91]	PROMs	Austria	Electronic	Single centre	40 children (35 aged 5-18 years and 5 proxies for children aged 1-4 years). all were white	Questions about pain, nausea, appetite loss, physical functioning, and sleep disturbance		Not stated speciality
Meyerheim 2022 [92]	PROMs	Germany	Electronic	Single centre		Self-report symptoms scales and diary entries		Multiple specialties
Morris 2023 [93]	PROMs	United Kingdom	Electronic	Single centre	Caregivers of CAMHS patients aged 4-18 years with a diagnosis of autism spectrum disorder (ASD). 98 completed the myHealthE and 98 completed the standard practice. 74% of children are males and mean age of 14.3	Strength and difficulties questionnaire- p for caregiver completion		Mental health
Munaretto 2021[94]	PROMs	Italy	Mixed	Single centre	18 caregivers and 50 patients 25% f with mean age of 16.4 years	Patient-reported outcomes measurement information system (PROMIS) questionnaire (parent proxy profile and pediatric profile)		Haematology
Musterd 2021 [97]	PROMs	Netherlands	Not stated	Single centre	120 patients aged 8-18 years	Juvenile arthritis multidimensional assessment report evaluation of quality of life (JQL) Checklist individual strength-8 (cis-9)		Rheumatology
Ndokera 2021 [171]	PREMs	United Kingdom	Not stated	Multicentre	67 carers		Focusing on experiences of attending the hospital and the impact of the necessary changes	Cardio, obstetrics neonatology, intensive, multiple
Ng 2023 [184]	PROMs and PREMs	Multiple countries	Mixed	Registry network	41 patient-parent dyads across 5 Starzl Network for Excellence in Pediatric Transplantation (SNEPT) sites for the first phase, 109 patient-parent dyads across 10 SNEPT sites for the second phase, 8 parents for phase 3 and 23 completed the PeltQL on a data-enabled FCC android device at one SNEPT site for phase 4. one year follow up in phase 1 and 2, only 2 of 10 SNEPT sites continued PeltQL administration	Pediatric liver transplant quality of life (PeltQL-self and proxy-versions)	User experience survey employed at various aspects of the implementation. all surveys to clinicians and parent stakeholders send via online survey platform	Transplant, multiple
Nordlind 2022 [172]	PREMs	Sweden	Mixed	Single centre			Variety of questionnaires used, some developed locally at the hospital whereas others were developed only for a specific department or ward in Electronic or paper formats. some were targeted to special patient group whereas others focused on experiences of hospitality participation and patients' satisfaction. Some had questionnaire only for children whereas other had just proxy or both	Not stated speciality
Saldana 2022 [116]	PROMs	United States	Not stated	Single centre	163 pediatric burn patients	Patient-reported outcomes measurement information system (PROMIS)		Respiratory
Santucci 2022 [119]	PROMs	United States	Not stated	Single centre	84 patients (aged 11-21 years, median age 16y, 71% female, 93% Caucasian)	Abdominal pain index (api Nausea severity scale (nss), Functional disability inventory (fdi) Pittsburgh sleep quality index (psqi) Children’s somatic symptoms inventory (cssi) Patient-reported outcomes measurement systems (PROMIS) Pediatric anxiety-short form Pediatric depression-short form scale		Pain
Santucci 2022 [119]	PROMs	United States	Not stated	Single centre	395 patients ages 8-18 years mean 14.82, 90% Caucasian and 76% females	Pain (pain intensity) Anxiety [screen for child anxiety related disorders (scared)] Patient-reported outcomes measurement systems (promis)-short form scales), Depression and/or suicidal ideation (phq-9)		Gastroenterology
Schlenz 2022 [127]	PROMs	Not stated	Not stated	Not stated	89 adolescents and young adults with sickle cell disease between ages 13-25	Ratings of sleep quality, overall pain, and 2 measures of pain related impairment (pain impact and pain burden).		Haematology
Schougaard 2019 [128]	PROMs	Denmark	Electronic	Single centre	Patients with epilepsy, 15 and over	Not stated		Neurology
Sheikh 2021 [131]	PROMs	United States	Pen and ppr	Single centre	21 patients aged between 13-25. 57% male. 17 completed first phase and 4 completed second phase	Adolescent md Anderson Symptom Inventory (mdasi-adol)		Oncology
Simpson 2020 [132]	PROMs	United States	Not stated	Single centre	233 patients had completed parent stress data, 44 had completed the GAD-7 and PHQ-9. mean age of 8.31, range between 2-20.4, 52.5% female	Parental stress scale (PSS) Pediatric Health Questionnaire (PHQ-9) General anxiety disorder (GAD-7)		Neurology
Smyth 2021 [133]	PROMs	Other	Electronic	Single centre	351 patients in the IBD program	The Pediatric Quality of Life Inventory version 4.0 short form generic core scale (PedsQL)		Gastroenterology
Stephens 2021 [174]	PREMs	United States	Electronic	Multicentre	7018 parents/guardians of children, 6584 were less than 18 years old	Press Ganey outpatient medical practice survey (PGOMPS)		Not stated speciality
Stratton 2022 [185]	PROMs and PREMs	Canada	Electronic	Single centre	PROMs-the 88 surveys analyzed belonged to 69 respondents, 32 were patients and 37 were parents/guardians. Of the 88 surveys, 42 were patients and 46 were parents/guardians. 56 were patient-parent dyads. Patients age ranged from 10-17 years	Patient and parent/guardian proxy metrics for pain location, duration, and intensity Relevant patient-reported outcomes measurement information system (PROMIS) metrics (pain intensity, sleep disturbance, pain interference, anxiety, depressive symptoms) Parent proxy short form anxiety and depressive symptoms Pain Catastrophizing Scale Child (PCS-C) and parent (PCS-P)	Post-questionnaire satisfaction survey to ascertain their priorities in the assessment of their transition pain	Pain
Swales 2022 [137]	PROMs	United Kingdom	Not stated	Single centre	8/13 children discussed in MDT	4 week seizure diaries 1 week diary of sleep, school attendance, and other activities, QOL questioners includes impact of epilepsy questionnaire and PedsQL		Rheumatology
Taxter 2022 [140]	PROMs	United States	Electronic	Single centre	58 patients with an incident diagnosis of jia during study period. 66% were female and 34% had oligoarticular juvenile idiopathic arthritis. Median age 10.8 years, between 6.5-14.9 years.	Pain Energy Patient disease activity Psychiatric symptoms Difficulties falling or staying asleep Patient global disease activity score part of CJADAS-10		Rheumatology
Teela 2019 [141]	PROMs	Netherlands	Electronic	Multicentre	8 children and 17 parents participated in the focus group	Not stated		Not stated speciality
Tyack 2020 [144]	PROMs	Australia	Electronic	Multicentre	30 interviews were conducted (14 children with life-altering skin conditions (burn scars, infantile hemangiomas and dermatological conditions), 16 clinicians). field notes involved 51 child and caregiver participants	Not stated but a measure of health-related quality of life		Dermatology
Vangorp 2021 [149]	PROMs	Netherlands	Electronic	Single centre	799 (caregivers of) children with cancer). Of these participating families, the mean age of the child was 9.4 and 55% was male. Hematological cancer was the most frequent diagnosis group (45% before treatment and 64% after treatment)	Dutch proxy- (2-7 years old) or self-report (age: 8-18 years) Pediatric quality of life inventory and Multidimension fatigue scales (PedsQL generic and fatigue scales) Distress thermometer for parents (DT-P)		Oncology
Vanmuilekom 2019 [150]	PROMs	Netherlands	Electronic	Not stated	Focus group - 8 adolescents (12-18 years) with chronic health conditions and parents (of children 0-18 years) registered on the KLIK website.	Questionnaires about health related quality of life (HRQOL)		Multiple speciality
Vanmuilekom 2021 [151]	PROMs	Netherlands	Electronic	Single centre	Children (8-12y) and adolescents (13-17y) from the general population (*n*=966) and from the pediatric population (*n*=1209)	PedsQL 4.0 (self-report) for children (8-12 years) and adolescent (13-17 years)		Multiple speciality
Vanmuilekom 2021 [152]	PROMs	Netherlands	Electronic	Not stated	28 clinicians participated (*n*=5) focus groups. Mean time in years as a KLIK user is 5.2 years with range of 0.3-7.4 includes medical doctor, psychologists, nurse or social worker	Patient-reported outcomes measurement information system (PROMIS)		Not stated speciality
VanOers 2021 [156]	PROMs	Netherlands	Electronic	Multicentre	Not stated	Health related quality of life		Multiple speciality
Vansonsbeek 2021 [187]	PROMs and PREMs	Netherlands	Electronic	Single centre	Adolescents aged 12-17 years old, and parents of children aged 4-17 years	Strength and difficulties questionnaire (SDQ) Kidscreen-52 child version and Kidscreen-27 parent version to measure child or adolescent's quality of life Treatment support measure (TSM), for parents (TSM-P) and youth (TSM-Y)	Youth thermometer- to measure satisfaction with the treatment they had received. the youth thermometer- child version consists of 28 items. the youth thermometer -parent versions ask about child's treatment and training in parenting skills (if applicable), so 31 or 32 items, respectively. subscales included appraisal information, appraisal participation, appraisal of the clinician (child's and parents' clinician), appraisal of treatment result and background information.	Mental health
Veltkamp 2022 [157]	PROMs	Netherlands	Electronic	Single centre	121 patients/parents registered themselves on the KLIK PROM portal. 112 completed at least one PROM	TNO-AZL preschool children quality of life (TAPQOL) for children under the age of 5, parents complete the questionnaires Pediatric Quality of life inventory (PedsQL), generic scale 4.0. Proxy reporting for children aged 2-7 years and self-reports for children aged 8-18 years		Nephrology
Vuong 2022 [159]	PROMs	Netherlands	Electronic	Single centre	109 children with media age of 11.8 years	Pediatric quality of life inventory		Haematology
Verkleij 2020 [158]	PROMs	Netherlands	Not stated	Single centre	15 primary ciliary dyskinesia patients aged 6-18years old	GAD-7 PHQ-9 QOL-PCD		Respiratory
Zachar-tirado 2021 [164]	PROMs	United States	Not stated	Single centre	101 children and adolescents with traumatic brain injury (mean age 15.4 years), 55% were male 88% were white	Physical health questionnaire (PHQ), 9 items (PHQ-9) and PHQ adolescent version (PHQ-A) and the short version of PHQ-A (PHQ-A-2)		Rehabilitation
VanOers 2013 [154]	PROMs	Netherlands	Electronic	Single centre	17 children with home parenteral nutrition (aged 8-18) (hpn) or their parents (children aged 0-7)	HRQOL		Gastroenterology
Vanoers 2018 [155]	PROMs	Netherlands	Electronic	Single centre	37 healthcare professionals (12 pediatricians, 14 nurses, 6 psychologists, 3 physiotherapists, 1 dietician, 1 social worker)	Not stated		Multiple speciality
Vangorp 2021 [149]	PROMs	Netherlands	Electronic	Single centre	799 pts (and their carers), mean age 9.4y (sd 4.9), 55% male	Dutch pedsql proxy or self-report		Oncology
Vandersluijsveer 2013	PROMs	Netherlands	Electronic	Not stated	Children with congenital hypothyroidism	Not stated		Endocrinology
Vandecrommert 2015 [147]	PROMs	Netherlands	Not stated	Single centre	Roughly 90 children with congenital adrenal hyperplasia (cah)	Qol tool		Endocrinology
Valles 2017 [146]	PROMs	Spain	Electronic	Multicentre	136 patients with type 1 diabetes (t1d) (72 girls, mean age 13.4y)	Kidscreen-27 (online)		Endocrinology
Uzark 2013 [145]	PROMs	United States	Not stated	Single centre	- 176 patients, aged 8.2-18.9years old (mean 12.8years old), 103m, 73m - 3 cardiologists			Cardiology
Townley 2019 [143]	PROMs	Canada	Not stated	Single centre	CYP aged 5-17	Self-report and proxy versions of the patient reported outcomes measures information systems (PROMIS)		Rehabilitation
Tollit 2019 [186]	PROMs and PREMs	Australia	Electronic	Single centre	Approx. 600 young people aged 3-17years old (prospective cohort)	Gender identity questionnaire Gender preoccupation and stability Questionnaire Gender slider Body image scale Social transition questionnaire Gender voice questionnaire Chest dysphoria scale Additional questions about effects of hormonal treatment designed by project team Youth self-report Short mood and feelings questionnaire Spence children's anxiety scale DASS21 Social phobia scale Gender minority stress and resilience measure Brief resilience scale Self-harm questionnaire Columbia-suicide severity rating scale Branched eating disorders test Social responsiveness scale-2 Psychological sense of school membership Gatehouse bullying scale Child health utility 9d (chu-9d) General functioning 12-item subscale of the McMaster family assessment device Questions adapted from communities that care survey and the childhood to adolescence transition study Items developed by the trans20 team and items based on the victorian population health survey		Not stated speciality
Spraggs-hughes 2018 [134]	PROMs	United States	Electronic	Multicentre	Not known	Varies according to population and subspecialties		Orthopaedic
Taxter 2018 [139]	PROMs	United States	Electronic	Single centre	615 clinic visits march-dec 2017	Questions about current symptoms, pain, fatigue, disease activity, function, and interest in research (in English and Spanish)		Rheumatology
Schepers 2013 [121]	PROMs	Netherlands	Not applicable	Single centre	118 patients/proxies; 31 clinicians	Generic PedsQL		Oncology
Schepers 2016 [123]	PROMs	Netherlands	Not applicable	Single centre	110 families	Physical/psychological ePROMs via KLIK		Oncology
Schepers 2016 [124]	PROMs	Not stated	Electronic	Single centre	29 clinicians	Not stated		Oncology
Scharrer 2013 [121]	PROMs	United Kingdom	Not stated	Single centre	50 pts randomly chosen under 12years with atopic eczema	Children’s dermatology life quality index (CDLQI) or infant’s dermatitis quality of life (IDQOL), plus pro forma to audit performance against nice guidelines		Dermatology
Segerdahl 2008 [129]	PROMs	Sweden	Pen and ppr	Multicentre	16,309 day surgeries in children 1-16years old	Visual analogue scale (VAS) Numerical scale rating (NRS) Wong scale		Surgery
Ryan 2013 [115]	PROMs	United States	Not stated	Single centre	112 pts aged 7-18years old (m=14.45, sd=2.92) with IBD and their parents	Pediatric quality of life inventory, version 4.0 (PedsQL 4.0) Children’s depression inventory: short version (CDI-S) Beck depression inventory second edition (BDI-II)		Gastroenterology
Romo 2016 [113]	PROMs	United States	Electronic	Single centre	Parents of 19 children aged 5-18years old	Burn outcomes questionnaire (BOQ - Plus pediatric symptom checklist (BOQ+P)		Burn
Robinson 2017 [111]	PROMs	United Kingdom	Pen and ppr	Single centre	All patients aged 5-19years (mean 13years) attending Sheffield children's hospital pain clinic for the first time between December 2009 and December 2014	Bath adolescent pain questionnaire (BAPQ) and version for parents (BAPQ-P) Daily functioning (physical and social) Emotional functioning (depression, general and pain related anxiety), Family functioning Development Only validated for age 11-18y but also used for 5-10y		
Robertson 2020 [109]	PROMs	United Kingdom	Not applicable	Single centre	18 clinicians completed survey, 27 took part in the focus group. clinicians are part of a multi-professional team comprising of opthalmologists, orthoptists, optometrists, clinical vision scientists, nurses and an eye clinic liaison officer	Vision-related quality of life (VQOL-CYP) Functional vision (FVQ-CYP)		Ophthalmology
Robertson 2020 [109]	PROMs	United Kingdom	Mixed	Single centre	93 pts aged 8-17years old, mean age 11years old (sd=2.4), 48 (52%) were male and 51 (55%) were white British. Forty-four (47%) were visually impaired and 10 (11%) were severely visually impaired or blind	Vision-related quality of life (VQOL CYP) and the functional vision (FYP CYP)		Ophthalmology
Robertson 2019 [108]	PROMs	United Kingdom	Not applicable	Single centre	18 clinicians			Ophthalmology
Richardson 2016 [105]	PROMs	United States	Not stated	Single centre	59 patients with acute kidney injury/also collected from other patients	HRQOL		Nephrology
Pennisi 2013 [101]	PROMs	Australia	Not stated	Multicentre	81 pts aged 14-18years old (mean 15years old sd 1.46), 34 male	Diabetes attitudes wishes and needs, monitoring individual needs in diabetes (dawnmind) youth questionnaire (my-q) who 5 well-being component		Endocrinology

**Fig. 2:**
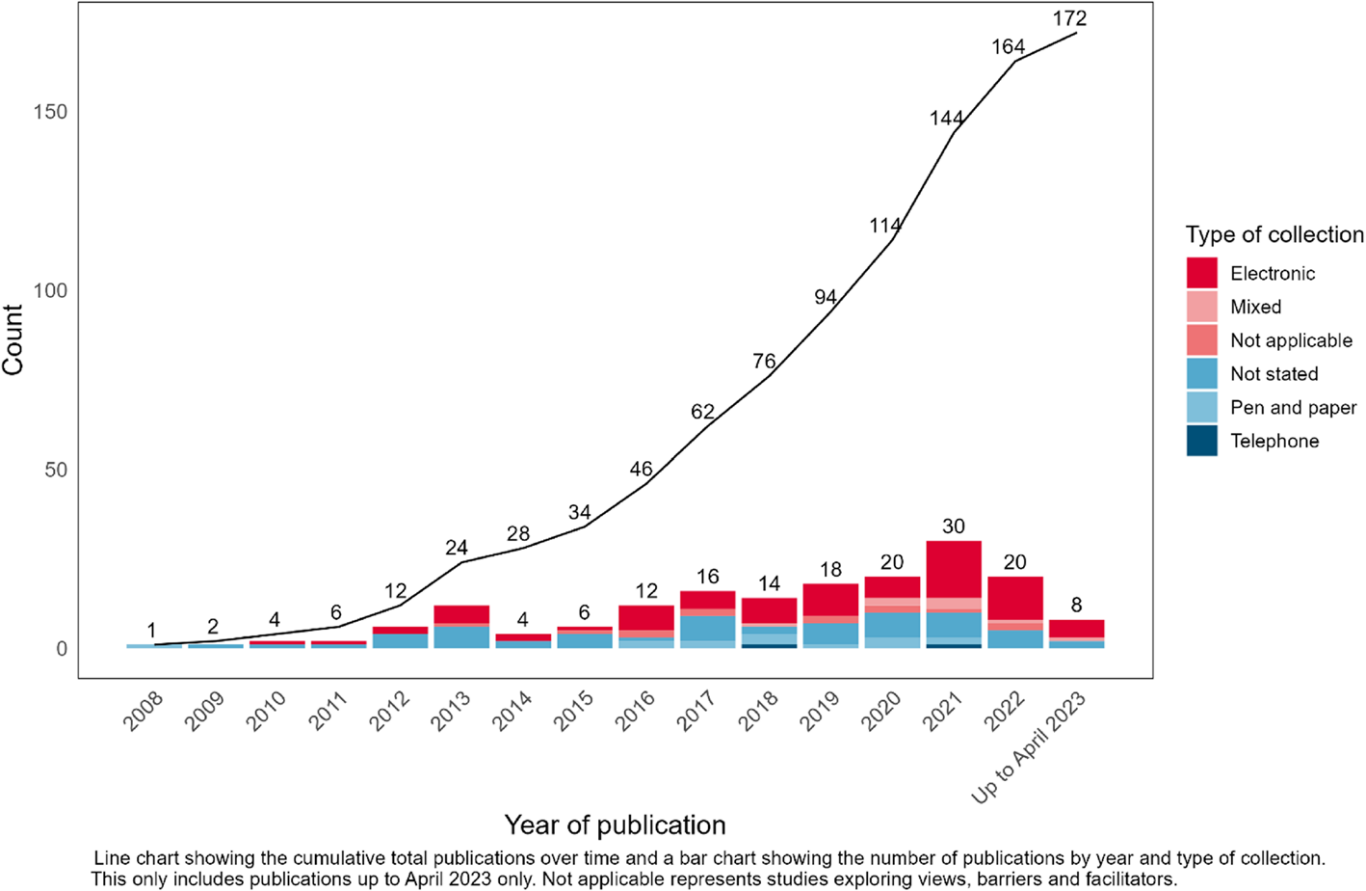
Number of new publications over time by type of collection

Most studies were conducted in one of four countries (USA, The Netherlands, UK and Canada), with a further 15 countries undertaking between one to five studies each. Amongst studies that collected PROMs/PREMs, 32% (*n*=56) did not report the type of collection, but the majority reported some form of electronic data capture (*n*= 80, 47%), such as the KLIK PROM portal, an online portal for PROMs, developed in the Netherlands (*n*=22, 13%). Figure 2 shows the increase in the number of publications per year regarding PROMs/PREMs, and also changes in method of collection over time.

Most sources did not state whether the hospital setting was inpatient and/or outpatient (*n*=80, 47%); however, amongst those that did, outpatient was the most common setting (*n*=73, 42%). A diverse range of individual specialties were represented, of which 28 different types were reported; oncology (*n*=17; 10%), endocrinology (*n*=12; 7%) and mental health (*n*=11; 6%) were the most frequently reported single specialities. A total of 120 (70%) reports described use of PREMs and/or PROMs in a single centre, 34 (20%) were multicentre and 13 (7%) either did not report the centre details or described data collection as part of standard care but then contributing to a registry or network (*n*=5, 3%).

Participants included CYP, parent/carer proxies and health professionals with numbers of participants varying from a single case study to 19,289 children. Participant groups and numbers were not always reported or were unclear.

### Which PROMs/PREMs were being used?

Some studies collected generic PROMs whereas other collected disease specific, symptom reporting, or psychosocial screening PROMs. The Pediatric Quality of Life Inventory (PedsQL) (*n*=42) was the most commonly collected and a range of non-validated measures of health-related quality of life were also used (*n*=14).

There were many different types of PREMs reported and only two studies used the same type of PREM (National Health Service patient survey). Five studies used PREMs that were either developed for a specific clinic, or department, or to evaluate a quality improvement initiative.

### How are PROMs/PREMs used to assess the experience and outcomes of CYP’s care and treatment in hospital?

Table 3 shows the summary of the themes in relation to all of the research questions. Full characteristics of included studies in relation to how PROMs/PREMs are used can be found in Additional File 5. The most common uses of PROMs/PREMs were for screening/monitoring to track patient outcomes and satisfaction longitudinally and to identify or detect problems/issues early [29, 34–36, 57–59, 62, 66, 70, 74, 76–78, 83, 86, 88–90, 94, 98, 109, 115, 133, 139, 141, 149, 156, 158, 161, 164, 166, 168, 174, 181, 184]. Some studies reported using PROMs/PREMs to assess changes in outcomes and experiences often associated with specific types of treatment or care plan [35, 39, 50, 69, 106, 109, 131, 137, 159, 168, 171–173, 177, 185, 187] whereas others used the results to provide further insight into QoL, functioning and/or symptoms that could be related to disease activity or outcomes [21, 24, 29, 49, 58, 85, 91, 116, 127, 140, 153]. PROM results were sometimes used to compare across other patient/population groups [32, 35, 51, 67, 74, 132, 133, 152]. PROMs/PREMs were also used in clinical encounters [9, 43, 61, 109, 141, 146, 150, 153, 155, 190] and as part of quality improvement initiatives [9, 37, 40, 96, 111, 172, 173, 175, 176] or to provide real-time advice or PROM/PREM results that could be incorporated within electronic health records [61, 134, 160]. This is particularly relevant for how the data are applied in clinical practice.

**Table 3:** Summary of themes in relation to the research questions by study type

**Summary of themes**	PROM (*N*, %)	PREM (*N*, %)	PROM and PREM (*N*, %)	Total (*N*, %)
**How are PROMs/PREMs used to assess the experience and outcomes of CYP’s care and treatment in hospital?**	76 (81%)	11 (12%)	7 (7%)	94
Screening/monitoring	31 (41%)	3 (27%)	2 (29%)	36 (38%)
Assessing treatment or care outcomes/changes in experience or care delivery	9 (12%)	4 (36%)	3 (43%)	16 (17%)
Insights into QOL/functioning/symptom and overall disease activity	12 (16%)			12 (13%)
Comparison with different population groups (proxies, other clinical groups, general population)	8 (11%)			8 (9%)
Used in clinical encounters	4 (5%)	4 (36%)	1 (14%)	9 (10%)
Quality improvement initiatives	3 (4%)			3 (3%)
EHRs available/results available prior to clinical encounters	9 (12%)		1 (14%)	10 (11%)
**How are PROM/PREM data applied in clinical practice?**	78 (87%)		12 (13%)	90
Help guide conversation and involve patients	16 (21%)		3 (25%)	19 (21%)
Tailored treatment	34 (44%)		5 (42%)	39 (43%)
Access to information	28 (36%)		4 (33%)	32 (36%)
**How do PROMs/PREMs contribute to service development?**	8 (67%)	1 (8%)	3 (25%)	12
Quality improvement/audits	4 (50%)	1 (100%)	1 (33%)	6 (50%)
Service planning	2 (25%)		1 (33%)	3 (25%)
Wider context	2 (25%)		1 (33%)	3 (25%)
**Are there any patient groups for whom PROMs/PREMs are not an integral part of routine care provision?**	26 (84%)	2 (6%)	3 (10%)	31
Organisational/health system constraints	11 (42%)	1 (50%)		12 (39%)
Measure restrictions	15 (58%)	1 (50%)	3 (100%)	19 (61%)
**What is the evidence on the availability and utilisation of reports generated from CYP and proxies?**	55 (69%)	5 (9%)	7 (10%)	67
Type of collection (if proxy only, or self-report only or both)	42 (76%)	5 (100%)	7 (100%)	54 (81%)
Proxies and self-reports provide different information	10 (18%)			10 (15%)
Privacy/ethical concerns	3 (6%)			3 (5%)

### How are PROM/PREM data applied in clinical practice?

PROM/PREM results can be directly applied in clinical practice to help guide or involve patients and their parents/carers in the conversation with clinicians and add information besides clinical judgement [9, 24, 31, 45, 46, 53, 56, 60, 68, 71, 76, 91, 99, 151, 153, 160, 165, 180, 183]. PROM/PREM results were also used to help tailor treatment plans, care pathways and referral to other services with patients’ goals [20, 24, 29, 33, 34, 48, 59, 61, 66–68, 73, 74, 76, 86, 89–92, 96, 98, 101, 104, 106, 131, 134, 141, 143, 145, 155, 156, 160, 161, 163, 182, 183, 186–188]. The results were also used to determine the frequency of contact with services, for example, telePRO use to determine timing of follow-up instead of fixed appointments or contact [67]. How data are applied in clinical practice was influenced by access to data. Some PROMs/PREMs were only reviewed during or before clinical encounters [47, 60, 63, 76, 79, 80, 104, 122, 124, 126, 134, 141, 142, 150, 152–154, 156, 157, 160, 163, 180, 181]. Others were analysed automatically, which triggered certain actions [21, 74, 86, 90, 91, 161, 188]—for example, if a threshold (a certain score) was reached, an automatic communication was sent to relevant clinicians (such as the primary clinician or location based social worker [86]. Storing PROM data in electronic health records to facilitate access and the availability of results was also described [21, 59, 74, 86, 186]. This was not restricted to those with electronic collection; for example Hacker et al. (2017) collected PROMs through pen and paper but scores and recommended interventions were documented in electronic health records [59]. PREM data were integral for quality improvement initiatives [172, 174, 185]. The full characteristics of the included studies can be found in Additional File 6.

### How do PROMs/PREMs contribute to service development?

Experiences or outcomes can be used as part of service planning, to decide on resource allocation [9]. Implementation of PROMs was seen to reduce use of services without compromising care [128]. Results from PROMs/PREMs collected in routine care can be used to identify changing needs of individual patients or patient cohorts [165], or as part of wider research initiatives for observational or translational research [165, 181], or to share best practices across health systems [102]. Overall, results are embedded as part of quality improvement projects or service audits, especially the use of PREMs where there is the ability to compare performance between services [9, 34, 96, 111, 150, 172]. The full description of the included studies can be found in Additional File 7.

### Are there any patient groups for whom PROMs/PREMs are not an integral part of routine care provision?

Many studies included in this review encompass the implementation stages, where the collection was part of standard clinical care, hence their inclusion. Furthermore, the common ‘bottom-up’ approach to implementation meant that routine collection was often for specific services [26, 43, 53, 63, 75, 134, 136, 154, 155], clinicians [108, 125] or areas [169]. However, in addition, there were also measure-specific restrictions for specific patient groups. Language was the most common restriction; for example, if patients did not speak or understand the majority language, they were not able to participate in the PROM/PREM data collection [23, 54, 86, 107, 126, 164, 176, 185, 187, 188, 190]. There were also other criteria for inclusion/exclusion which could reduce representation of the patient cohort, such as not including CYP if orthopaedic surgery was on the dominant hand or only including CYP with severe skin disease [25, 29, 40, 75, 78, 164], cognitive and/or developmental abilities in the normal range [34, 54, 77, 86, 131, 185] or those in a particular age group [54, 75, 185]. The full description of the included studies can be found in Additional File 8.

### What is the evidence on the availability and utilisation of reports generated from CYP and proxies?

Thirty-seven studies reported both proxy and self-reports [21, 25, 27, 33, 36, 45, 46, 54, 60, 63, 65, 72, 75, 80, 81, 84, 88, 98, 107, 115, 121, 125, 139, 142, 143, 145, 149, 161, 170, 180, 182, 186]. There were also some situations where either proxy reports [45, 70, 73, 79, 80, 95, 98, 112, 113, 160, 161, 163, 167, 169, 175–177, 188, 189] or patient reports only were standard [86, 117, 136]. Other studies only used proxies in certain situations, such as for younger children (typically under 7 years) [46, 79, 80, 124, 125, 149, 154, 170, 180, 181]. Parents and clinicians also sometimes helped children complete the measures [76, 88].

Having both proxy and self-reports can result in discrepancies in reporting [50, 51, 54, 55, 75, 83, 106, 110]. Parents sometimes felt that they needed to correct the children’s responses [110], and often clinicians viewed the proxy reports as adding information to the measures that could sometimes be more accurate than that from children’s reports [22, 48, 50, 75, 110]. However, this can create complexity in consultations if clinicians are unclear on which reports to use [33] and when to disclose results [150], especially if the child sees the measures as sharing secret information with the clinician [76]. The full description of the included studies can be found in Additional File 9.

### What are the barriers and facilitators to the routine utilisation of PROMs/PREMs in hospital care for CYP?

**Fig. 3:**
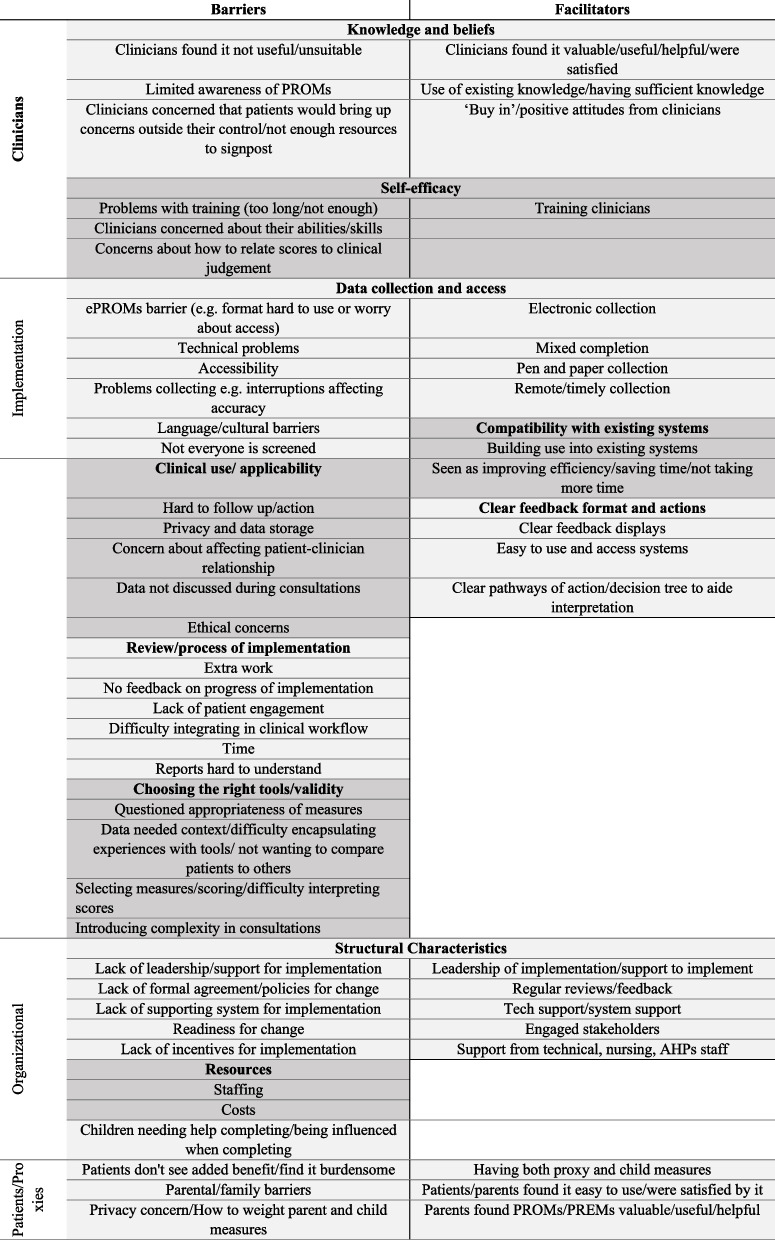
Barriers and facilitators to routine use of PROMs and PREMs in the care of CYP

Figure 3 shows the symmetry between barriers and facilitators in relation to clinicians, the implementation process, organisational processes and patients/proxies. Key barriers relevant to pediatric care included privacy concerns, specifically patients ‘providing false information if their parents could access their […] score’ [190] or concerns of how to ‘discuss PROs in the presence of parents/child’ [150]. McCabe et al. [9] further suggested having ‘separate portals for parents and caregiver [….] to give the clinician the ability to control which pieces of information are available to parents and children’. Furthermore, children might sometimes need help from their parents to complete the measures, which could influence their scores [23, 33, 110]. Having both proxy and child measures was a key facilitator [9, 33].

## Discussion

### Key findings

This review sought to understand the use and benefits of PROMs/PREMs collected within routine care of CYP in hospitals. Overall, PROMs are more commonly used than PREMs. These measures are generally collected using electronic methods and there are specific specialties, settings, context and countries that are leading the efforts to embed them in their routine collection and use. Results of PROMs/PREMs can be applied in clinical practice and/or service development, but whether and how this is done are not consistently reported. The use of PROMs/PREMs gives CYP, and their families/carers, the opportunity to be involved in their care and to help tailor treatment/care decisions based on their perspectives. However, specific challenges remain in the use of PROMs/PREMs for CYP that need to be considered in their routine implementation.

The use of PROMs/PREMs with CYP introduces complexities due to children’s varying age, cognitive and developmental abilities and the addition of proxy measures. This review demonstrates that both self and proxy measures tend to be collected and that children sometimes need help completing their own measures, but children and young people have their own individual perspectives which should be heard. This can generate specific barriers such as privacy issues of sharing and influencing the results between children, young people and their parents/carers and practical issues such as how to resolve discrepancies between reports. In addition, there is the relative weight that clinicians may give to CYP and proxy reports from adult caregivers. Careful consideration is needed when choosing the most appropriate measures and access to results and further support may be needed to understand how to use and discuss or weight self and proxy reports as part of standard care for CYP [9]. As such, it is important to explore context-specific factors within any organisation as this could influence the potential barriers encountered and effective facilitators needed to address these [10].

To our knowledge, this is the first review to explore the use of both PROMs and PREMs. We demonstrate similarities in how PROMs/PREMs are used but, ultimately, they are distinct in how they contribute to different aspects of routine care. PROMs can be directly applied to clinical practice to help guide and add to the consultation or tailor care, whereas PREMs are integral for quality improvement initiatives and facilitate comparisons between services. The synchronous use of PROMs/PREMs can facilitate a comprehensive capture of the patient perspective [3, 5]. However, this review also found that the routine use of PREMs is far less commonly reported than the routine use of PROMs in the literature, the reasons for which are beyond the scope of this review. This is consistent with the existing literature in pediatric care [191–193] and may reflect the lack of understanding of what PREMs are, their value, and how they can facilitate quality improvement initiatives [193]. Additionally, views on implementing and valuing PREMs in pediatric care warrant further exploration.

The findings of this review mirror findings in recent reviews showing that PROMs in pediatric care settings can be used to identify CYP’s needs, facilitate better communication and lead to improvements in the quality of care [7, 10]. We now extend this by demonstrating the evolving landscape of the routine implementation in the pediatric care setting. There is growing interest and progress in the implementation of routine collection of PROMs but this scoping review highlights that this is skewed towards single centres, specific specialties, settings and countries. Consequently, the current bottom-up approach to implementation suggests that more progress is needed to make routine collection and use of PROMs more accessible across different contexts, specialties, settings and countries. This also includes understanding how to make the results interoperable across different health systems. These steps may be important in healthcare providers and policymakers recognising the potential role and value of using PROMs and PREMs in routine care, to support scalability and wider implementation. Furthermore, it was noticeable that representation from low- and middle-income countries was poor.

Previous evidence recommends the use of electronic measures for CYP [2]; this is reflected in the findings of this review whereby PROMs/PREMs were predominantly collected through electronic measures. This is mostly led by specific systems such as the KLIK PROM portal; however, such standalone systems can be expensive and resource intensive to implement. Therefore, it is unclear whether a widespread adoption of electronic collection can be sustained across different settings and health systems. Additionally, the scoping review found that ‘pen and paper’ or mixed methods of data collection can also act as a facilitator, which raises the question of suitability of preference for electronic collection only. Similarly, the review also identified incorporation of PROMs/PREMs data into electronic health records as a facilitator and that this aids the availability and use of results. The integration of PROMs/PREMs into electronic health records is not always guaranteed for electronic collection and can also happen for those collected via pen and paper. Consequently, this suggests the need for further understanding of what is meant by electronic PROMs/PREMs and whether electronic collection and capture of results are viewed as interchangeable. Furthermore, the popularity of electronic collection and capture warrants further investigation on how to capitalise on these technological advancements to facilitate meaningful use of PROMs/PREMs in routine practice.

### Strengths and limitations

By exploring PROMs/PREMs together in pediatric hospital care, we offer insights on the extent to which outcomes and experiences of CYP and their families/carers are collected and utilised to inform their care. This scoping review was explorative in nature; it included an array of sources including conference proceedings from a variety of conditions, settings, contexts and countries and allowed us to extract information from all sections of the publication [19], which meant that this review was able to capture an extensive view of the existing literature. Furthermore, this review represents searches from two adjacent time periods, thereby demonstrating the changing nature of this field, whilst also facilitating further analyses of how terms have changed over time [194].

As this is a scoping review, the quality of evidence was not evaluated; therefore, we cannot comment on the risk of bias. Furthermore, it is unclear to what extent potential bias can arise from the high number of publications from certain groups, hospitals or countries. Similarly, due to the nature of routine collection, some studies reported different aspects of the same cohort; therefore, this may overrepresent routine collection. In addition, this scoping review did not include studies not published in the English language and it was sometimes difficult to conclude whether PROMs/PREMs were routinely collected and how they were used in routine practice. These may contribute to an underrepresentation of the available evidence. Furthermore, it is highly likely that there is an underreporting of routine use of PROMs/PREMs, particularly the latter, and results may not always be published. More comprehensive and systematic reporting is needed on the routine collection and use of PROMs/PREMs in the treatment and care of CYP to fully understand and leverage the potential benefits. Finally, we did not register the protocol on a public database, but we do appreciate that this is now best practice for scoping reviews, as well as systematic review protocols.

## Conclusion

PROM/PREM data have the potential to improve the quality of care of children and young people in hospitals but introduce specific challenges that need to be considered in their implementation as part of standard care. PROMs/PREMs contribute to different aspects of patient care but the potential for PREMs to improve patient care may be underutilised. More progress has been made on the routine use of PROMs; however, this is concentrated within specific specialties, settings, contexts and countries. Better understanding is needed of the use of PROMs/PREMs and of how findings are being applied to routine care, particularly with electronic collection and capture, to enable meaningful use.

## Supplementary Information

Supplementary Material 1.

## Data Availability

Majority of the data extracted from the studies and analysed in the review are included in this published article (Table 2) and its additional files (Additional files 5–9). The categorisation of themes and data extract is available on Mural (https://app.mural.co/embed/e708d9c9-3f9d-4de5-8606-7e11e7ed86de) and the codes used for the categorisation of the frequency counts are available on GitHub (https://github.com/HFAnalyticsLab/PROMs_PREMs_in_CYPs_Scoping_Review).

## Abbreviations

PROMs: Patient-reported outcome measures
PREMs: Patient reported experience measures
CYP: Children and young people
QoL: Quality of life
