# Supplementary material for: Routine use of patient-reported experience and outcome measures for children and young people: a scoping review
Source: Syst Rev. 2024 Nov 28;13:293. doi: 10.1186/s13643-024-02706-x (PMC11603634; doi:10.1186/s13643-024-02706-x)
Supplement: Supplementary file 1 — Supplementary Material 1. [file 13643_2024_2706_MOESM1_ESM.docx]

**Additional File 1- Full inclusion and exclusion criteria**

**Additional File 2: Final search terms with an example of search strategy**

**Additional File 3: The extraction form**

**Additional File 4: Full summary statistics.**

**Additional File 5: Summary of key themes and characteristics of included studies on how PROMs and PREMs are used in treatment and care of CYPs.**

**Additional File 6: Summary of key themes and characteristics of included studies on how PROMs and PREMs data are applied in clinical care.**

**Additional File 7: Summary of key themes and characteristics of included studies on how PROMs and PREMs contribute to service development.**

**Additional File 8: Summary of key themes and characteristics of included studies on patient groups for whom PREMs and PROMs are not an integral part of routine care provision.**

**Additional File 9: Summary of key themes and characteristics of included studies on the evidence on the availability and utilisation of reports generated from CYP themselves and proxy reports (from parents).**

Additional File 1: Full inclusion and exclusion criteria

| Inclusion Criteria | Exclusion Criteria |
| --- | --- |
| Population  CYP from birth to 25 years old, or their proxies, treated in paediatric settings (including neonatal and adolescent).  If transitioning from children to adult services, patients are included if they are still in the paediatric setting. | Population  CYP treated in adult hospital settings (inpatient or outpatient)  Adults (aged over 25) |
| Concept  PROM and/or PREM use including:   - Friends and family test (FFT) - QOL/HRQoL measures - Disease-specific measures | Concept   - Research trials where PROMs/PREMs data are outcomes measured – e.g., trials/studies where PROMs and/or PREMs are measured in non-routine care - Developing or validating PROM or PREM tool |
| Context  Routine clinical care for CYP in paediatric hospital care (including neonatal) inpatient or outpatient service within an adult hospital or children’s hospital, including:   - Medical and surgical - Emergency care (including in an emergency department providing paediatric services within an adult hospital) - Transition care (in paediatric setting) - Intensive care - Neonatal care - Paediatric outreach - Virtual paediatric hospital clinics - Specialist adolescent and young adult services (inpatient and outpatient) where data from patients under the age of 25 years can be separated from those over the age of 25 years. | Context   - Primary care - Adult emergency department setting with no paediatric service or professional input. - Adult inpatient or outpatient settings - Schools - Homes (unless part of paediatric outreach or virtual paediatric hospital clinics) - Hospices - Maternity services (including those caring AYAs) - Community clinics - Dental care |

Additional File 2: Example of search strategy (Medline)

| # | Searches | Results |
| --- | --- | --- |
| 1 | patient reported outcome measures/ | 7520 |
| 2 | PREM*.mp. | 377661 |
| 3 | (experience adj measure).mp. | 109 |
| 4 | ((friends and family test) or FFT).mp. | 2822 |
| 5 | (PROMs or PROM or PROMIS).mp. | 7006 |
| 6 | (patient reported adj (outcome* or experience* or treatment outcome* or indicator*)).mp. | 25341 |
| 7 | (patient adj (outcome assessment* or outcome measure* or survey* or questionnaire*)).mp. | 10789 |
| 8 | patient cent* outcome measure.mp. | 52 |
| 9 | patient experience questionnaire.mp. | 78 |
| 10 | (Self-report* adj (measure* or outcome*)).mp. | 15488 |
| 11 | (parent-report* adj (measure* or outcome* or experience*)).mp. | 659 |
| 12 | (quality of life or QoL or health-related quality of life or HRQoL or HRQL or health status).mp. | 495900 |
| 13 | 2 or 3 or 4 or 5 or 6 or 7 or 8 or 9 or 10 or 11 or 12 | 904901 |
| 14 | 1 or 13 | 904901 |
| 15 | Pediatrics/ | 54962 |
| 16 | adolescent/ or exp child/ or exp infant/ | 3628448 |
| 17 | (child* or adolescen* or teen* or youth* or young person* or young people* or infan* or toddler* or baby or babies).mp. | 4189308 |
| 18 | p?ediatric*.mp. | 427591 |
| 19 | 15 or 16 or 17 or 18 | 4254091 |
| 20 | 14 and 19 | 267046 |
| 21 | (clinical care or clinical practice or clinical implementation or clinical service* or clinical team*).mp. | 230335 |
| 22 | exp Patients/ | 68138 |
| 23 | (hospital patient* or inpatient* or outpatient*).mp. | 299696 |
| 24 | 22 or 23 | 328372 |
| 25 | 21 or 24 | 549106 |
| 26 | 20 and 25 | 11441 |
| 27 | 26 not clinical trial.pt. | 10951 |
| 28 | limit 27 to (english language and yr="2008 -Current") | 7559 |
| 29 | exp Animals/ | 23826038 |
| 30 | Humans/ | 19036693 |
| 31 | 29 not 30 | 4789345 |
| 32 | 28 not 31 | 7551 |

Additional File 3: Extraction form

| Types of evidence source |  |
| --- | --- |
| Evidence source Details and Characteristics | |
| Citation details |  |
| Author/s |  |
| Year of publication |  |
| Country |  |
| Method of PROMs and PREMs collection |  |
| Participants (details e.g., age/sex and number) |  |
| Details/Results extracted from source of evidence | |
| PREM/s used |  |
| PROM/s reported (including tool details) |  |
| Context/setting (e.g. ward, emergency care) |  |
| Key findings in relation to:   1. How PROMs and PREMs are used. 2. How PREMs and PROMs data applied in clinical practice. 3. Barriers and facilitators. 4. Data utilised towards the development of health care services. 5. Patient groups for whom PROMs and PREMs are/are not an integral part of routine care provision. 6. Reports from CYP themselves vs proxies (parent/carer). 7. Data capture and access of ePROMs/ePROMs results for different demographic breakdowns. |  |

Additional File 4: Full summary statistics.

| **Characteristics** | **N = 172** (N, %) |
| --- | --- |
| **Collection Type** |  |
| Electronic | 80 (47%) |
| Mixed | 6 (33%) |
| Pen and paper | 16 (9%) |
| Telephone | 2 (1%) |
| Not applicable | 12 (7%) |
| Not stated | 56 (32%) |
| **Type** |  |
| PROMs | 149 (86%) |
| PREMs | 11 (6%) |
| PROMS and PREM | 14 (8%) |
| **Participant** |  |
| Patient | 51 (30%) |
| Proxy | 12 (7%) |
| Patient and proxy | 45 (26%) |
| Clinician | 23 (13%) |
| Clinician and proxy | 10 (6%) |
| Patient, proxy and clinician | 17 (10%) |
| Not stated/Not clear | 11 (6%) |
| **Setting** |  |
| Outpatient | 73 (42%) |
| Inpatient | 13 (8%) |
| Mixture | 6 (3%) |
| Not stated | 80 (47%) |
| **Speciality** |  |
| Cardiology | 4 (2%) |
| Mental health | 11 (6%) |
| Surgery | 2 (1%) |
| Oncology | 17 (10%) |
| Palliative | 1 (1%) |
| Respiratory | 8 (5%) |
| Burn | 5 (3%) |
| Dermatology | 7 (4%) |
| Psychology | 2 (1%) |
| Psychiatry | 2 (1%) |
| Nephrology | 3 (2%) |
| Neurology | 6 (3%) |
| Rheumatology | 7 (4%) |
| Haematology | 10 (6%) |
| Gastroenterology | 6 (3%) |
| Ophthalmology | 3 (2%) |
| Endocrinology | 12 (7%) |
| Orthopaedic | 5 (3%) |
| Immunology | 1 (1%) |
| Pain | 5 (3%) |
| Urology | 2 (1%) |
| Occupational therapy | 1 (1%) |
| Transplant | 8 (5%) |
| Rehabilitation | 5 (3%) |
| Transition/Gender services | 2 (1%) |
| Allergy | 1 (1%) |
| Multiple | 11 (6%) |
| Not stated | 26 (15%) |
| **Context** |  |
| Multicenter | 34 (20%) |
| Single Centre | 120 (70%) |
| Registry Network | 5 (3%) |
| Not Stated | 13 (8%) |
| **Country** |  |
| Argentina | 1 (1%) |
| Australia | 5 (3%) |
| Austria | 5 (3%) |
| Canada | 14 (8%) |
| Denmark | 3 (2%) |
| Finland | 1 (1%) |
| France | 1 (1%) |
| Germany | 2 (1%) |
| India | 1 (1%) |
| Italy | 1 (1%) |
| Netherlands | 39 (23%) |
| New Zealand | 1 (1%) |
| Norway | 1 (1%) |
| Singapore | 1 (1%) |
| Spain | 1 (1%) |
| Sweden | 3 (2%) |
| Turkey | 1 (1%) |
| United Kingdom | 26 (15%) |
| United States | 53 (31%) |
| Multiple countries | 5 (3%) |
| Other | 1 (1%) |
| Not stated | 6 (3%) |

Additional File 5: Summary of key themes and characteristics of included studies on how PROMs and PREMs are used in treatment and care of CYPs

| Author | Wide Theme | Theme | Key findings |
| --- | --- | --- | --- |
| Gupta 2023 | Screening/Monitoring.  Insights into QOL/Functioning/Symptom and/overall disease activity. | Regular/longitudinal reporting. Insights into functioning/symptoms/QOL. Screening to identify problems. | -ESAS implemented for screening to improve supportive care and achieve symptom control.  -Scores were used to explore symptom prevalence, severity and trajectory. |
| Haverman 2017 | Screening/Monitoring. | Screening to identify problems. Regular/longitudinal reporting. | -Implementation of KLIK in various sites and specialities.  -KLIK aims to monitor and screen children over time to detect problems early and intervene.  -Generic and disease specific PROMs are available, children and parents can complete PROMs, stage and frequency of completion are determined by the clinical team.  -Patients and parents are invited to join by letter and register on the KLIK website where they receive an email asking them to complete PROMs. |
| Riedl 2022 | Assessing treatment or care outcomes/ changes in care delivery and overall experience. | Assessing treatment/surgical/care/Rehabilitation outcomes. | -PROMs used to assess rehabilitation outcomes.  -Collected before being admitted and at the end of rehabilitation stay. |
| Bjertnaes 2018 | Assessing treatment or care outcomes/ changes in care delivery and overall experience. | Evaluate changes/experiences. | -PREM developed to test three data collection models (electronic-only, paper-only, both electronic and paper). |
| vanMuilekom 2022 | Insights into QOL/Functioning/Symptoms and/overall disease activity.  Used in clinical encounters. | Insights into functioning/symptoms/QOL. Discussed during consultations. | -PROMs collected through the creation of KLIK account, answers are converted into KLIK ePROfiles which clinicians discuss during consultations.  - KLIK provides insight into their daily functioning and improves conversation content during the consultation where a broader range of topics is discussed. It can also help patients prepare for the consultation.  -Mixed views on the added value and goal of KLIK. |
| Taxter 2022 | Insights into QOL/Functioning/Symptoms and/overall disease activity.  Assessing treatment or care outcomes/ changes in care delivery and overall experience. | Association with other clinical outcomes.  Assessing treatment/surgical/care/Rehabilitation outcomes. | -PROMs administered prior to provider visits and were integrated into electronic health records.  - PROMs used to assess the association between outcomes and Juvenile Idiopathic Arthritis treatment and whether symptom duration prior to diagnosis is associated with disease activity scores over time. |
| vanMuilekom 2021 | Comparisons with different groups. | Comparing with general population. | -PROMs collected through KLIK PROMs portal.  -Study compare PedsQL from paediatric population to general population. |
| Smyth 2021 | Comparisons with different groups.  Screening/Monitoring. | Comparing with different population.  Screening to identify problems. | -Comparing results from Canada to different populations (of chronic illness, age, healthy controls) across the world. They identified studies from EMBASS and OVID since 2003. I-n the specific IBD program, PROMs was used identify and support IBV patients with psychosocial issues associated with their disease. |
| Simpson 2020 | Comparisons with different groups. | Comparing with self and proxies. | -Measuring parent-reported stress for parents of children and children with spinal defects and whether the two are related. |
| Hames 2016 | Used in clinical encounters.  EHR available prior to clinical encounters. | Tailored care/shared decision making.  EHR available prior to clinical encounters. | -Integrating Mental and Physical Healthcare: Research, Training and Services (IMPARTS) web-based screening system is used to collect patient-reported outcomes that can help provide real-time, tailored referral advice. |
| Teela 2019 | Used in clinical encounters.  Screening/Monitoring. | Help with consultation or communication.  Screening to identify problems. | -PROMs used to monitor well-being over time, detect psychosocial or HRQOL problems or symptoms early.  -Parents value KLIK as it helps them prepare for the consultations, assist them to talk about psychosocial functioning and leads to more efficient consultation.  -Patients/parents completed PROMs online at home prior to outpatient consultation. |
| McCabe 2023 | Quality improvement initiatives.  Used in clinical encounters. | Quality improvements.  Tailored care/shared decision making.  Service Audit. | -PROMs and PREMs were used to improve care at individual level by enhancing clinicians' understanding of patient as a whole and facilitating shared decision making.  -PROMs and PREMs were also as indicators of quality of care to make improvements to services, decisions about resource allocation and demonstrate value of programs.  -PROMs are also sometimes mandated by funders and can be used for research purposes (e.g., clinical research, registries). |
| Devereux 2009 | Quality improvement initiatives. | Service Audit. | -PROMS used as part of service audit.  -Recommendations to give CDLQI for all new patients. |
| Wray 2019 | Quality improvement initiatives. | Service planning. | -Data from PREMs analysed and SMART plans developed for each team.  -Summary of findings and improvements reported to parents in next cycle. |
| Spraggs-Hughes 2018 | EHR available prior to clinical encounters. | EHR available prior to clinical encounters. | -All PROMIS measures are given in computer adaptive testing (CAT) format and results are delivered in real time to the Electronic medical record (EMR) |
| vanGorp 2021 | Screening/Monitoring. | Regular/longitudinal reporting. Screening to identify problems. | -PROMs used for regular psychosocial monitoring and screening.  -Using KLIK PROMs portal. |
| Gupta 2021 | Screening/Monitoring. | Screening to identify problems. | -PROMs used to screen for common symptoms and assist providers in supportive care and symptom control. |
| Fischmeister 2021 | Assessing treatment or care outcomes/ changes in care delivery and overall experience. | Assessing treatment/surgical/care/Rehabilitation outcomes. | -HRQOL used to assess treatment success during an inpatient rehabilitation stay. |
| Holzman 2021 | Assessing treatment or care outcomes/ changes in care delivery and overall experience.  Screening/Monitoring. | Evaluate changes/experiences.  Regular/longitudinal reporting. | -Exploring patient satisfaction with telemedicine during the COVID-19 pandemic. |
| Cheng 2022 | Comparisons with different groups. | comparing with general population. | -All PROMIS domains are normalised to the general US population.  -PROMs are correlated with social deprivation. |
| Dalton 2022 | Comparisons with different groups.  Screening/Monitoring.  Assessing treatment or care outcomes/ changes in care delivery and overall experience. | Comparing with different population.  Regular/longitudinal reporting. Assessing treatment/surgical/care/Rehabilitation outcomes. | -PROMs used to collect longitudinal surgical outcomes data for individual patients and used to compare outcomes for different diagnostic groups and track changes with patients’ age over time.  -Collection of PROMs had minimal interference to the smooth and efficient running of the clinic. |
| Franklin 2021 | Comparisons with different groups. | comparing with self and proxies. | -Assess whether parents/caregivers give the same perception of their child's function as the child themselves. |
| Bele 2022 | Used in clinical encounters. | Tailored care/shared decision making. | -Used PROMs to understand the overall impact of clinical condition on the patient and provide comprehensive care aligned with patients' goals.  -PROMs capture patients' and family member's perspectives in a standardised manner. |
| VanMuilekom 2019 | Used in clinical encounters. | Discussed during consultations. | -Discussed PROs during their consultations. |
| Robertson 2020 | Used in clinical encounters.  Assessing treatment or care outcomes/ changes in care delivery and overall. experience. Screening/Monitoring. | Help with consultation or communication.  Assessing treatment/surgical/care/Rehabilitation outcomes.  Regular/longitudinal reporting. Screening to identify problems. | -According to patients PROMs would be useful when making clinical decisions, detecting problems and concerns that clinical assessments would not identify, monitoring a patient’s condition and response to treatment.  -PROMs would also be useful for improving communication and join-decision making with patients and their families. |
| Wray 2020 | Quality improvement initiatives. | Quality improvements. | -PREMs used as part of quality improvement cycle across different specialities. |
| Robinson 2017 | Quality improvement initiatives. | Service Audit. | -PROMs used to conduct a service audit. |
| Pryde 2021 | Quality improvement initiatives.  Assessing treatment or care outcomes/ changes in care delivery and overall experience. | Service planning.  Evaluate changes/experiences. | -PREMs used to evaluate families' experiences and inform service planning and design moving forward. |
| Wang 2018 | EHR available prior to clinical encounters. | EHR available/ results available prior to clinical encounters. | -PROMs results available to clinicians prior to a patient’s encounter via a real-time delivery. |
| Kuhn 2022 | Screening/Monitoring.  Comparisons with different groups. | Regular/longitudinal reporting. Comparing with different population | Implementation study that evaluates the impact of PEESS v20 into monitoring of Eosinophilic Esophagitis (EoE) disease status. Comparing PEESS patient’s vs non PEESS |
| Mentrikoski 2018 | Screening/Monitoring. | Screening to identify problems. | -PROMs used as psychosocial screening tool. |
| Vuong 2022 | Assessing treatment or care outcomes/ changes in care delivery and overall experience. | Assessing treatment/surgical/care/Rehabilitation outcomes. | -PROMs used to evaluate the impact of hospital admission on health-related quality of life. |
| Ndokera 2021 | Assessing treatment or care outcomes/ changes in care delivery and overall experience. | Evaluate changes/experiences. | -PREMs used to assess necessary changes during COVID-19 such as remote meetings, use of personal and protective equipment and social distancing. |
| Meryk 2022 | Insights into QOL/Functioning/Symptoms and/overall disease activity.  Screening/Monitoring. | Insights into functioning/symptoms/QOL.  Regular/longitudinal reporting. | -PROMs used to monitor daily symptom burden.  -Web-based PROMS monitoring to enable real-time symptom monitoring by the healthcare providers. |
| Schlenz 2022 | Insights into QOL/Functioning/Symptoms and/overall disease activity. | Association with other clinical outcomes. | -PROMs used to assess sleep quality in relation to Pain and Pain-related impairment in adolescents and young adults with sickle cell disease |
| Valles 2017 | Used in clinical encounters. | Discussed during consultations. | -Discussed HRQOL results with doctor quarterly over 1 year. |
| Nordlind 2022 | Quality improvement initiatives. Assessing treatment or care outcomes/ changes in care delivery and overall experience. | Quality improvements. Service planning.  Evaluate changes/experiences. | -PREMs used as children's voices on their health care experiences. Also used for strengthening children’s participation and autonomy.  -PREMs often used to assess quality improvement work such as identifying the needs and request of different groups of patients, comparing results with other departments, patients’ safety work, educating staff and customizing auxiliary areas e.g., waiting room, managing adverse events and care facilities. |
| Carberry 2016 | Screening/Monitoring. Insights into QOL/Functioning/Symptoms and/overall disease activity. | Regular/longitudinal reporting. Insights into functioning/symptoms/QOL. | -PROMs used as part of initial patient intake and for ongoing assessment of disease burden.  -Disease burden score is automatically calculated. |
| Hacker 2017 | Screening/Monitoring. | Screening to identify problems. | -Depression and suicidal ideations (SI) screening implemented in 2016 aiming to screen all patients > 12 years old during annual social work visits.  -Paper tool given at start of visit and hand scored. |
| Swales 2022 | Assessing treatment or care outcomes/ changes in care delivery and overall experience. | Assessing treatment/surgical/care/Rehabilitation outcomes. | -PROMs collected at baseline, 3 months and 6 months post treatment to explore the effectiveness of evorolimus. |
| Stratton 2022 | Assessing treatment or care outcomes/ changes in care delivery and overall experience. | Evaluate changes/experiences. | -Incorporating patient and family feedback during clinic appointments using questionnaires.  -Satisfaction surveys were used to evaluate this change and make changes were appropriate. |
| Hjollund 2023 | Comparisons with different groups. | comparing with different population | -PROMs used to highlight specific telePRO solutions for follow up, describe the algorithm outcomes and variation in outcomes.  -Results were also used to state the similarities and difference between patient groups. |
| VanOers 2018 | Used in clinical encounters. | Discussed during consultations. | -PROMs used in over 50 patient groups.  -Results transformed into ePROfiles and clinicians discusses them with the patients. |
| Dharmaraj 2019 | Quality improvement initiatives. | Quality improvements. | -PeLTQL has been incorporated into clinical practice since 2013 and is used as part of quality improvement project. |
| Taxter 2018 | Screening/Monitoring. | Regular/longitudinal reporting. | -Completed prior to or at clinic visit. Providers can trend longitudinal data during a clinic visit using flowsheet functions which can also be shared with patients. Also used to identify patients interested in research. |
| WheatButt 2014 | Screening/Monitoring. | Screening to identify problems. | -Annual psychosocial screening for all patients as part of routine care. |
| Mandell 2021 | Insights into QOL/Functioning/Symptoms and/overall disease activity. | Association with other clinical outcomes. | -PROMs used to assess relationship between quality of life and burn injury related factors |
| Eilander 2016 | Used in clinical encounters. | Discussed during consultations. | -Adolescents complete MIND Youth-Questionnaire (MY-Q) annually online before consultation and outcomes are discussed with patients. |
| Murphy 2017 | Quality improvement initiatives. | Quality improvements. | -Used quality-improvement methodology to integrate use of EPP-QOL at annual review. |
| Ng 2023 | Screening/Monitoring. | Regular/longitudinal reporting. | -QoL assessment and monitoring in long-term ambulatory surveillance care of paediatric LT survivors. |
| Naranjo 2017 | Screening/Monitoring. | Screening to identify problems. | -PROMs used for mental health screening. |
| Henning 2022 | Screening/Monitoring. | Screening to identify problems. | -PROMs used for screening to optimise individual patient care, detection of hidden comorbidity and for facilitation of research. |
| Dhar 2021 | Assessing treatment or care outcomes/ changes in care delivery and overall experience. | Assessing treatment/surgical/care/Rehabilitation outcomes. | -Used to assess treatments for addressing atopic dermatitis. |
| Bele 2022 | Insights into QOL/Functioning/Symptoms and/overall disease activity. | Insights into functioning/symptoms/QOL. | -PROMs would help them with a holistic understanding of patients' and families' needs including psychosocial aspects impacting their health status.  -PROMs can also play a role in patient empowerment by capturing patients' and family member's perspectives in a standardised way. |
| Saldana 2022 | Insights into QOL/Functioning/Symptoms and/overall disease activity. | Association with other clinical outcomes. | -PROMs used to measure the impact of Burn injuries on quality of life. |
| Maasalo 2017 | Screening/Monitoring. | Regular/longitudinal reporting. | -SDQ collected as part of routine clinical baseline measurement. |
| Cunningham 2018 | Screening/Monitoring. | Screening to identify problems. | -Used as routine screening tools for anxiety, Pain and Pain-related disability.  -Data captured directly into EMR. |
| Jalilova 2023 | Assessing treatment or care outcomes/ changes in care delivery and overall experience. | Assessing treatment/surgical/care/Rehabilitation outcomes. | -Comparing psychological characteristics of T1D patients and parents started on a-HCL. |
| Hanmer 2021 | Screening/Monitoring. | Screening to identify problems. | -Questionnaires used as screening, can be completed in a tablet or if not completed can be completed through Electronic Health Records by asking the patient or parent verbally. |
| vanSonsbeek 2021 | Assessing treatment or care outcomes/ changes in care delivery and overall experience. | Assessing treatment/surgical/care/Rehabilitation outcomes. | -Using questionnaires to indicate which aspects of the treatment is working. |
| Fischer 2020 | Insights into QOL/Functioning/Symptoms and/overall disease activity. | Association with other clinical outcomes. | -Looking at association between HRQL and HbA1c levels |
| Lewer 2011 | Screening/Monitoring. | Regular/longitudinal reporting. | -PedsQL used as part of clinical practice at all visits and tracked longitudinally. |
| Sheikh 2021 | Assessing treatment or care outcomes/ changes in care delivery and overall experience. | Assessing treatment/surgical/care/Rehabilitation outcomes. | -Used MDASI-ADOL to assess symptom burden before and after HSCT and to improve outcomes. |
| VanGorp 2021 | Screening/Monitoring. | Regular/longitudinal reporting. | -The psychosocial functioning of children with cancer and their caregivers were assessed as part of regular monitoring, but this study compared pre and post COVID-19. |
| Ryan 2013 | Screening/Monitoring. | Screening to identify problems. | -PROMs used for psychosocial screening. |
| Aldekhyyel 2018 | Insights into QOL/Functioning/Symptoms and/overall disease activity. | Insights into functioning/symptoms/QOL. | -New Pain Management Interface (PMI) which patients/parents can use to report Pain assessments at pre-defined times according to hospital's nursing Pain reassessment policy. |
| Munaretto 2021 | Insights into QOL/Functioning/Symptoms and/overall disease activity.  Screening/Monitoring. | Association with other clinical outcomes. | -Correlating QoL with clinical haematological and therapeutic variables. |
| Marker 2019 | Screening/Monitoring. | Screening to identify problems. | -Annual depression screening implemented at four clinics |
| Meryk 2021 | Screening/Monitoring. | Regular/longitudinal reporting. | -ePROtect monitors patients' symptom burden during and after treatment. |
| Leahy 2021 | Screening/Monitoring. | Regular/longitudinal reporting. | -Feasibility of collecting patient-reported symptom monitoring in hospitalised paediatric patients with cancer for routine clinical care. |
| DaRocha 2020 | Screening/Monitoring. | Screening to identify problems. | -Screening pilot programme from February 2017-February 2019 asking patient-parent pairs to complete PROMS. |
| Verkleij 2020 | Screening/Monitoring. | Screening to identify problems. | -Mental health screening during regular yearly outpatient visits. |
| Kermarrec 2015 | Screening/Monitoring_ | Screening to identify problems. | -All pts under 5 years old are screened. |
| Stephens 2021 | Screening/Monitoring. | Regular/longitudinal reporting. | -To measure and track patient satisfaction scores in outpatient encounters. |
| Zachar-Tirado 2021 | Screening/Monitoring. | Screening to identify problems. | -Screening instrument for depressive disorders in adolescents with traumatic brain injury to determine the sensitivity of a shorter screening tool" |
| Caddell 2015 | Screening/Monitoring. | Regular/longitudinal reporting. | -Used to check for trends in patient satisfaction |
| Lassen 2023 | Screening/Monitoring. | Screening to identify problems. | -Used to screen for disturbed eating behaviour (e.g., skipping meals and feelings of shame associated with eating) to prevent eating disorders and disease-related complications. Questionnaires completed 7 days prior to their consultation either at home or in the waiting room. |
| vanOers 2021 | Screening/Monitoring. | Screening to identify problems. | -Patients complete PROMs online prior to visit, answers are transformed into ePROfiles, clinicians discuss this ePROfile with patients to monitor well-being over time, identify problems and provide tailored advice and interventions. Seen as a valuable tool for monitor PROMs in their patients. |

Additional File 6: Key themes and characteristics of includes studies on how PROMs and PREMs results are applied in clinical practice.

| Author | Wide Theme | Theme | Key findings |
| --- | --- | --- | --- |
| Lassen 2023 | Help guide conversation and involve patients, Access to information, Tailor treatment | Guide conversation, Discussed or used during clinical encounter, Referral to additional services follow up | -On the basis of PROMs and clinical assessment, patients could be offered supplementary multidisciplinary treatment by a psychologist, psychiatrists, nurse, paediatrician, and/or/ dietician.  -PROMs could also led to supplementary treatments such as counselling sessions with psychologists.  - PROMs can identify problems, help clinicians prepare for the consultations, help participants guide the conversation to their views and overall promote and improve consultations. |
| Haverman 2013 | Help guide conversation and involve patients, Access to information | Involve patients, Discussed or used during clinical encounter | -When clinicians were provided with ePROfiles and this was discussed during consultation, there was more discussion of emotions and family. |
| Zia 2016 | Help guide conversation and involve patients | Added information | -Surveillance and analysis of health outcomes such as quality of life, fatigue, etc are integrated into care model for clinical care. |
| Wolfe 2014 | Tailor treatment,  Access to information | Referral to additional services follow up, Referral to additional services follow up, Set actions after specific threshold/trigger, Tailored advice aligned with patient goals, Immediate assessment of results | -Oncologists and families received PQ reports and emails, for clinicians this was before the visit, for families this was immediately after survey completion.  -The reports included trends in results, summary highlighting changes since last report, list of available resources for symptom control and generic pain management recommendations.  -Reports contributed sometimes to clinician’s decision to initiate psychosocial, pain, social work or palliative care and consulting and discussing goals with the families.  -PQ-emails were automatically generated for certain criteria. These emails went to providers (oncologists, nurse, psychosocial clinician), the palliative care services and when pain was reported the pain service. |
| Haverman 2017 | Access to information | Discussed or used during clinical encounter, Viewed before clinic encounters, Presented outside clinical workflow | - Answers from PROMs collated into ePROfiles available for patient, parent and team to view on a website. Results can be presented before, during or after consultation. Team members can only see results relevant to them.  -For some patient groups, results are discussed in multidisciplinary team meeting. |
| Hacker 2017 | Access to information, Tailor treatment | Results stored in EHRs, Set actions after specific threshold/trigger | -Scores and recommended interventions were documented in the electronic health record.  -Patients who flagged positive for SI completed additional risk assessment and level of care needed was determined immediately. |
| vanMuilekom 2021 | Help guide conversation and involve patients, Access to information | Guide conversation, Discussed or used during clinical encounter | -Clinicians indicated that feedback of individual items is essential for the use of PROMs in clinical practice.  -Clinicians use the items to start a dialogue (as a conversation tool), to understand the domain scores that are provided and to discuss specific problems. |
| McCabe 2023 | Help guide conversation and involve patients | Involve patients | -Improve care at the individual level by enhancing clinicians' understanding of patient as a whole and facilitating shared decision making |
| Bele 2022 | Help guide conversation and involve patients, Tailor treatment | Added information, Tailored advice aligned with patient goals | -KidsPRO programme is an ehealth solution that supports and facilitates integration of PROMs into routine paediatric clinical care).  -Participants believed that implementing PROMs would not drastically change the current practice of providing care but rather enhance it.  -Participants suggested that using PROMs would help them understand the overall impact of the clinical condition on the patient and provide comprehensive care aligned with patients' goals. |
| Townley 2019 | Tailor treatment | Referral to additional services follow up | -As appropriate, patients were referred for further investigation as identified through the tool and clinical expertise. |
| vanSonsbeek 2021 | Tailor treatment | Set actions after specific threshold/trigger, Tailored advice aligned with patient goals | - Clinicians tracks whether a patient is on track or not using SDQ scores.  -There were different interventions if a patient is not in track, including providing practical suggestions about how the treatment might be improved which is discussed with the patient or their parents to determine how to get the treatment back on track. Another intervention includes also discussing the feedback with a colleague.  -Through a discussion with the patient, clinician can be more responsive to deterioration and modify treatment when needed to reduce treatment failures. |
| Wang 2018 | Access to information, Help guide conversation and involve patients, Tailor treatment | Viewed before clinic encounters, Guide conversation, Tailored advice aligned with patient goals | - Scores calculated and summary presented to clinicians before the patient’s clinic appointment or after the outpatient surgery.  -BOQ+P data stimulated conversation with the patient or family member and the information impacted the interventions clinicians recommended during the encounter. |
| Kuhn 2022 | Access to information, Tailor treatment | Immediate assessment of results, Tailored advice aligned with patient goals, Set actions after specific threshold/trigger, Results stored in EHRs | -An automated myGeisinger message was sent to patients scoring above this threshold and epic staff message to the Geisinger paediatric gastroenterology nursing pool in basket for internal tracking.  -The message to patients acknowledges the increase in symptoms, reinforced treatment adherence and provide instructions to contact the Geisinger paediatric gastroenterology if with questions or to request a follow up appointment if needed.  - The completed EOE peessv2.0 responses were visible to the physician care team in each patient's epic EMR synopsis as a flow sheet which can be viewed at any time. The results could also be populated into epic progress notes by 'dotphrase' (eoesurveyresults). |
| Marker 2019 | Access to information,  Tailor treatment | Results stored in EHRs, Immediate assessment of results, Set actions after specific threshold/trigger, Tailored advice aligned with patient goals | -Real time data listening and automated processes which reviews PROMs data and pulls data to EHRs.  -Scores on the depression items of the PHQ-2 fires a real-time automatic trigger allowing the patient to complete the PHQ-9 within RedCap to gather additional symptom information and an automatic alert to primary clinician and the location-based social worker. |
| Gerhardt 2018 | Help guide conversation and involve patients | Guide conversation,  Involve patients, Added information | -PROMs usage shifted the dialogue between patients and clinicians and now view this as an opportunity to ask more about self-management. |
| Kliems 2020 | Help guide conversation and involve patients | Added information | -Some providers thought that the family relationships measure provided information they currently do not have, that it provides a more efficient and systematic way to get information or serve as a conversation aid for the clinical encounter.  - More interested in seeing individual scores than summary scores. |
| Henning 2022 | Tailor treatment | Referral to additional services follow up, Tailored advice aligned with patient goals | -PROMs assisted in deciding with ASM to choose referral to specialist/multidisciplinary team reduce taboo around psychiatric comorbidity and facilitate discussion. |
| Meryk 2022 | Tailor treatment, Help guide conversation and involve patients, Access to information | Set actions after specific threshold/trigger, Involve patients, Immediate assessment of results,  Tailored advice aligned with patient goals, Referral to additional services follow up | - For inpatients symptom monitoring was checked daily before the morning round and if the threshold was met were reported then results were discussed immediately at the patient’s bedsides.  -For outpatients symptom monitoring was checked daily by the medical team and if the threshold was met then patients or their caregivers were immediately called by the physicians to confirm the reported symptoms, discuss the situation, and initiate an intervention if needed. |
| Mims 2019 | Tailor treatment, Help guide conversation and involve patients | Tailored advice aligned with patient goals, Added information | -Identification of some patient issues that might not have surfaced, physician documentation of PRO responses and when appropriate, interventions. |
| Veltkamp 2022 | Access to information | Discussed or used during clinical encounter | -The clinician discusses ePROfile with patient and/or parents during outpatient visits. |
| Spraggs-Hughes 2018 | Access to information, Tailor treatment | Viewed before clinic encounters,  Tailored advice aligned with patient goals | - Clinicians can view results before appointment.  - Clinicians have formulated formal handouts for patients to address heightened anxiety and/or depression scores at the time of clinical care. |
| Aldekhyyel 2018 | Access to information | Immediate assessment of results, Results stored in EHRs | - Data from pain assessment is immediately communicated to nurse's phone and documented in EHR. nurses then reassess.  - System also links patient/parent to non-pharmacological resources. |
| Griffiths 2017 | Help guide conversation and involve patients | Guide conversation, Involve patients | -Outcomes data are incorporated into formulation meetings and clinical reviews, aiding reflections on individual’s progress. |
| Tollit 2019 | Tailor treatment, Access to information | Referral to additional services follow up,  Tailored advice aligned with patient goals, Results stored in EHRs | -To assist clinicians in providing patient care. PROMs responses are uploaded onto EMR and are available to treating team to guide assessment and treatment.  -Clinician’s follow-up as deemed clinically relevant. |
| Katsicas 2011 | Tailor treatment | Tailored advice aligned with patient goals | - Interdisciplinary team tailored strategy for each patient. |
| Ng 2020 | Help guide conversation and involve | Guide conversation  Added information | -Uncovered PROM issues not otherwise raised during the visit. |
| Hall 2014 | Help guide conversation and involve patients, Access to information | Involve patients, Guide conversation, Discussed or used during clinical encounter | -HCPs used reports to engage with patients and to expand discussions with young people who struggled to communicate. Used to open channels of communication between hcp, family and pt. |
| Cox 2021 | Tailor treatment | Referral to additional services follow up | -Different for different clinicians, but includes quickly checking areas of concern, and making referrals based on scores. |
| Teela 2019 | Tailor treatment, Access to information | Tailored advice aligned with patient goals,  Discussed or used during clinical encounter | -The HCPs discusses KLIK ePROfile during consultation and provide tailored advice and interventions. |
| Schepers 2017 | Access to information | Discussed or used during clinical encounter | -Discussed ePROfiles. |
| WheatButt 2014 | Access to information, Tailor treatment | Immediate assessment of results, Set actions after specific threshold/trigger | -Anxiety and depression measures are scored immediately and clinical elevation of either score automatically assessed and/or referred. |
| Cunningham 2018 | Tailor treatment | Referral to additional services follow up, Tailored advice aligned with patient goals | -Medical providers received an automated PROMs to tailor care, including to consider psychological referral. |
| vanOers 2021 | Tailor treatment Access to information | Tailored advice aligned with patient goals,  Discussed or used during clinical encounter | -Clinicians discuss this ePROfiles with patients to monitor wellbeing over time, identify problems and provide tailored advice and interventions. |
| Huang 2012 | Help guide conversation and involve patients,  Tailor treatment | Involve patients, Tailored advice aligned with patient goals | -To evaluate treatment effectiveness, developing treatment plan, and involving patients in the consultation process. |
| Chen 2022 | Help guide conversation and involve patients | Added information | -PRO assessment provides more benefits to patients than relying on clinical judgement alone. |
| Fernandez-Quintana 2021 | Tailor treatment | Set actions after specific threshold/trigger | -With the new cutoff points, ASRS-A and ASRS-P can be considered a clinical tool in the screening and diagnosis of adolescents referred to CAMHs due to suspected ADHD. |
| Schepers 2014 | Access to information | Discussed or used during clinical encounter | -Data transformed into profiles and clinicians were trained to interpret the profile and discuss with patients/parents in 3 consecutive follow up appointments. |
| vanMuilekom 2022 | Help guide conversation and involve patients, Access to information | Involve patients, Discussed or used during clinical encounter | -Clinicians discuss KLIK ePROfiles with patients and parents during consultation, but this doesn't always happen/doesn't happen enough according to parents in which case some parents discussed the PROMs themselves.  -More topics were discussed using KLIK. |
| Racine 2018 | Tailor treatment, Access to information | Tailored advice aligned with patient goals,  Discussed or used during clinical encounter | -PROMs used to monitor symptoms and determine when a psychiatry consultation was needed specifically in relation to medication.  -PROMs were discussed at the beginning of each session and compared in relation to previous weeks. PROMs ere used to discuss readiness for termination. |
| Engelen 2012 | Help guide conversation and involve patients, Access to information | Guide conversation, Discussed or used during clinical encounter | -Emotional and psychosocial functioning was discussed more intensively and more emotional and cognitive problems were identified when using PROMs. |
| Bele 2022 | Help guide conversation and involve patients | Involve patients | - PROMs to help with understanding patients' and families' needs, including psychosocial aspects.  - PROMs can also help empower patients. |
| Sheikh 2021 | Tailor treatment | Referral to additional services follow up | -PROMs used to refer to additional services such as physical therapy, occupational therapy, dietitian, supportive care/palliative care, psychology/psychiatry and ancillary services (child life services or music therapy. It was also used to start or adjust for medications such as pain or antiemetic medication. |
| Schepers 2016 | Access to information | Discussed or used during clinical encounter | -ePROfiles are discussed during consultations. |
| Engelen 2010 | Help guide conversation and involve patients | Guide conversation | - QLIC-ON profile presented to paediatric oncologist and helps to endure that psychosocial functioning is systematically addressed during consultations. |
| VanMuilekom 2019 | Access to information | Discussed or used during clinical encounter | -Discussed PROMs during consultations. |
| Naranjo 2017 | Tailor treatment | Referral to additional services follow up | -Positive screening were followed up within 6 months and referred to attend behavioural health appointment. |
| Meryk 2021 | Tailor treatment | Set actions after specific threshold/trigger, Referral to additional services follow up | - When symptom severity of level 3 or 4 was reported, an automated alarm to health care team was triggered by the ePROtect system and interventions were undertaken such as immediate counselling about symptom management with direct consultation for inpatients and via telephone for patients in home care, extension of supportive medication and recommendation for prompt clinical presentation and/or admission for patients in home care.  -The patient was contacted by the health care team when combinations of moderate system resulted in significant symptom burden and has persisted over a period of 48-72h. |
| Kroupina 2020 | Tailor treatment | Tailored advice aligned with patient goals | -Based on the results of the assessment, patients and their parents are assigned to an interventional model. |
| Meyerheim 2022 | Tailor treatment | Referral to additional services follow up | -Qualitative observations of single cases show that EPROs can be useful for diagnostic purposes and other therapeutic interventions. |
| Yao 2019 | Tailor treatment, Access to information | Set actions after specific threshold/trigger, Discussed or used during clinical encounter | -Parents reported QoL discussed in clinical practice.  - A clinical decision support was developed to direct referral to a diabetes educator for QOL scores. |
| Limperg 2013 | Access to information | Discussed or used during clinical encounter | Data converted into KLIK ePROfile and discussed during consultation with paediatric haematologist. |
| Hames 2016 | Tailor treatment | Referral to additional services follow up | -All patients screening positive for probable major depressive disorder (PMDD) or probable generalized anxiety disorder (PGAD) were offered a referral to the clinical psychologist. |
| Pennisi 2013 | Tailor treatment | Set actions after specific threshold/trigger | -Total score of 13 or less warrants further investigation |
| Teela 2020 | Access to information | Discussed or used during clinical encounter | -Answers are converted into a KLIK ePROfile, which is discussed by the paediatrician during consultation. |
| Murphy 2017 | Tailor treatment | Set actions after specific threshold/trigger, Tailored advice aligned with patient goals | -Enabled team to identify and provide assistance to a patient in crisis. |
| Hjollund 2023 | Tailor treatment | Referral to additional services follow up | -TelePRO uses fixed questionnaires as the basis for follow up instead of fixed appointments and contact. Patient pathways in PRO algorithm-based follow up. |
| Riedl 2022 | Tailor treatment | Tailored advice aligned with patient goals | -As required by the Australian health insurance pension fund, individual rehabilitation goals are discussed with patients at the beginning of the treatment and outcomes is evaluated by the healthcare professionals and patients based on these goals. |
| VanOers 2013 | Access to information | Discussed or used during clinical encounter | -Answers converted into ePROfiles and paediatricians discusses these during consultation. |
| Limperg 2012 | Access to information | Discussed or used during clinical encounter | -Converted into KLIK ePROfile and discussed in routine care.  -SDQ discussed with psychologist. |
| VanOers 2018 | Tailor treatment | Tailored advice aligned with patient goals | -Tailored interventions provided. |
| Carberry 2016 | Tailor treatment | Tailored advice aligned with patient goals | -Clinicians and patients together referenced the scores from prior visits and used the functional assessment information to guide interventions and escalations of care. |
| Uzark 2013 | Tailor treatment | Tailored advice aligned with patient goals | - Complete PROMs were either placed in patient’s chart or given directly to clinician by family.  - Clinician reviews responses that are “often” or “almost always” answers and document the action taken in response to the identified problems in a form of checklist or in the letter to the child’s primary care providers. |
| Aberdeen 2019 | Tailor treatment | Tailored advice aligned with patient goals | -PROMs data used to assess response to treatment, inform clinical prognosis, guide clinical decisions and satisfy insurance requirements. |

Additional File 7: Key themes and characteristics of includes studies on how PROMs and PREMs contribute to service development.

| Author | Wider Theme | Theme | Key findings |
| --- | --- | --- | --- |
| Haverman 2017 | Wider context | Research initiatives | - Patients and parents were asked for permission to use PROMs data for research.  - Evaluation questionnaire was filled out for KLIK. |
| Perito 2021 | Wider context | Sharing best practice | -STARZL network run as a learning health system (LHS) bringing together patients, families and providers to identify challenges and improve and PROs are incorporated into outcome metrics. The aim is to identify and share best practices across centers. |
| Mccabe 2023 | Service planning, Quality improvement or audits | Resource allocation, Help clinicians improve | -Enhancing quality of care- data can be useful for making improvement to their services and help make decisions about resource allocation.  -PROMs and PREMs are also seen as a feedback tool for own clinicians' personal growth and beneficial in advocating for additional services for certain populations because they can demonstrate the burden of those conditions. |
| Schougaard 2019 | Service planning | Lower use of services | -Slightly fewer telephone consultations and slightly lower emergency room visits when using PROMs. |
| Zia 2016 | Service planning, Wider context | Identify changing needs of patients, Research initiatives | -Surveillance and analysis of health outcomes integrated into comprehensive multidisciplinary care model but also observational and translational research initiatives.  -Plan to use this information critically to identify the changing needs of the patients, identifying problems that need to be addressed and to document the effect that our healthcare delivery model has made. |
| Nordlind 2022 | Quality improvement or audits | Quality improvement, Empower patients | -PREMs often used to assess quality improvement work such as identifying the needs and request of different groups of patients.  -Also used for strengthening children's participation and autonomy, improving information for children, improving information for custodians, comparing our results with other departments, patients' safety work, clarifying children's position and integrity, educating the staff, improving the possibilities for play and occupation, customizing auxiliary areas, e.g., waiting room, managing adverse events, and customizing care facilities. |
| Vanmuilekom 2019 | Quality improvement or audits | Empower patients | -To help inform the development of educational videos to empower paediatric patients and parents to discuss PROS and optimize the communication between patients/parents and the HCP. |
| Robinson 2017 | Quality improvement or audits | Quality improvement | -Used as part of audit of service. |
| Murphy 2017 | Quality improvement or audits | Quality improvement | -Part of QI project. |
| Cunningham 2018 | Quality improvement or audits | Quality improvement | -6-month QI project using plan-do-study-act framework |

Additional File 8: Key themes and characteristics of includes studies on any patient groups for whom PROMs/PREMs are not an integral part of routine care.

| Author | Wider Theme | Theme | Key findings |
| --- | --- | --- | --- |
| Eilander 2016 | Organisational health systems constraints | Only specific services clinics departments | -Integrated into 2 of the 10 clinics |
| Robertson 2019 | Organisational health systems constraints | Depending on clinicians | -78% clinicians not using PROMs routinely for paediatric ophthalmology. |
| Kemp 2018 | Organisational health systems constraints | Depending on area | -Routine in 3 provinces in Canada |
| Vansonsbeek 2021 | Measure restrictions | Not fluent or not speaking specific language | -Having insufficient understanding of the Dutch language |
| Zachar-tirado 2021 | Measure restrictions | Clinical restrictions, Not fluent or not speaking specific language | -if not fluent in English, orthopaedic injury to the dominant hand |
| Stratton 2022 | Measure restrictions | Cognitive abilities, Age, Developmental ability, Not fluent or not speaking specific language | -Questionnaires were not sent to patients who were developmentally younger than 8 years old or to patients or parents/guardians who were known to the pedTPS as not being fluent in English regardless of developmental age. |
| Leahy 2021 | Measure restrictions | Developmental ability, Cognitive abilities | Inclusion criteria includes being developmentally and cognitively capable of self-reporting and being English literate. |
| Haverman 2013 | Organisational health systems constraints | Only specific services clinics departments | -Implemented in follow-up care |
| Schepers 2016 | Organisational health systems constraints | Depending on clinicians | -Not used by majority of HCPs. |
| Wray 2020 | Measure restrictions | Not fluent or not speaking specific language | -Not available in languages other than English |
| Carberry 2016 | Measure restrictions | Clinical restrictions | -Before intervention no surgical subspecialties besides the congenital heart and transplant surgery groups conducted prospective patient outcomes measurement but by 2015, the outcomes of over 1300 unique patients with supracondylar fractures, cleft lip and/or palate, or voiding dysfunction had been tracked. |
| Gmuca 2019 | Measure restrictions | Age, Cognitive abilities, Not fluent or not speaking specific language | -Patients excluded if they were non-English-speaking, intellectual disabilities, younger than 8 years old. |
| Bower 2020 | Organisational health systems constraints | Only specific services clinics departments | -Not currently routine - aim to increase to 70% of paediatric palliative care encounters. |
| Wolfe 2014 | Measure restrictions | Not fluent or not speaking specific language | -Non English speaking parents excluded |
| Kuijlaars 2019 | Measure restrictions , Organisational health systems constraints | Clinical restrictions, Age, Only specific services clinics departments | -Since 2010, ped-HAL is part of routine assessment of severe/moderate haemophilia patients aged 4-18 treated at the Van Creveldkliniek in Utrecht. |
| Sheikh 2021 | Measure restrictions | Cognitive abilities | -Those under 13 or unable to comprehend or speak English well enough to understand the questions and patients with altered mental status were excluded from administration. |
| Vanoers 2013 | Organisational health systems constraints | Only specific services clinics departments | -Children with KLIK-PROs programme at Emma Children's Hospital |
| Schepers 2017 | Measure restrictions | Not fluent or not speaking specific language | -Only available for those who understand Dutch well enough to complete the PROMs. |
| Dharmaraj 2019 | Measure restrictions | Clinical restrictions | -Routinely used for LT paediatric patients - but not clear how routine use has been sustained or what happens to the data. |
| Marker 2019 | Measure restrictions | Cognitive abilities, Not fluent or not speaking specific language | -Over 2 years across 4 clinics, screening rates using the PHQ-2/PHQ-9.  -Patients excluded if they could not complete PROM e.g., non-English speaking, developmental delay |
| Swales 2016 | Organisational health systems constraints | Only specific services clinics departments | -Four adolescent DBT programmes were routinely collecting data with sufficient accuracy to benchmark. |
| Lewer 2011 | Measure restrictions | Clinical restrictions | -CYP with atopic dermatitis routinely assessed. |
| Cunningham 2018 | Measure restrictions | Cognitive abilities | -Children with developmental delays not included |
| Blaauboer 2017 | Measure restrictions | Clinical restrictions | -CYP with severe skin disease in this setting complete PROMs as part of routine care. |
| Vanoers 2018 | Organisational health systems constraints | Only specific services clinics departments | -Since the start of the KLIK implementation in 2011, 50 patient groups (e.g. rheumatology, oncology) use KLIK in 20 Dutch hospitals, 700 healthcare professionals are trained and 10, 000 patients are registered. |
| Gerhardt 2018 | Organisational health systems constraints | Only specific services clinics departments | -Some departments now use PROMs routinely. |
| Barthel 2016 | Measure restrictions | Not fluent or not speaking specific language | -Children who do not speak/write German. |
| Spraggs-hughes 2018 | Organisational health systems constraints | Only specific services clinics departments | -In summer 2015, the orthopaedic outpatient clinics of an academic medical centre implemented the collection of PROMIS assessments. As of 2018, PROMIS assessments are collected for the outpatient clinical visits of 60 orthopaedic faculty and 17 midlevel providers across 7 separate locations in a metropolitan area (adult and paediatric). |
| Bele 2022 | Measure restrictions | Not fluent or not speaking specific language | -Language barriers: reading of non-English speaking population could create a language barrier. |
| Riobueno-naylor 2019 | Measure restrictions | Not fluent or not speaking specific language | -Not available for parents not literate in English or Spanish. Not yet routine in this setting. |

Additional File 9: Key themes and characteristics of includes studies on the evidence and utilisation of reports generated from CYP and proxies?

| Author | Wider Theme | Theme | Key findings |
| --- | --- | --- | --- |
| Darocha 2020 | Type of collection | Both used | -CYP and parents both took part. |
| Naranjo 2017 | Type of collection | Proxy standard, Both used | -Patients over 12 completed own PROM; patients under 12 did not (unless parent had positive screening). |
| Schepers 2016 | Type of collection | Proxy only for certain criteria, Both used | -HCPs preferred child and parent report for 8-12y and 13-17y and 18-21y. |
| Marker 2019 | Type of collection | Patient reported only | -Measures completed by CYP population. |
| Swales 2016 | Type of collection | Clinicians completed,  Patient reported only | -Most young people scored questionnaires themselves.  -Some clinicians completed as proxies. |
| Mentrikoski 2018 | Type of collection | Parents helped completing self reports, Both used | -Patients over 4 years old completed self-reported with support if needed. Caregivers of children aged 0-17 years completed caregiver questionnaires. |
| Riedl 2022 | Proxies and self reports provide diff info | Discrepancies between reports | -Children and their parents showed substantial discrepancies in their reports. |
| Fischmeister 2021 | Proxies and self reports provide diff info | Proxies provide additional info, Discrepancies between reports | -Parents reported medium to large differences in more than half of the assessed symptoms but the children did not report significant improvements.  -Parent proxy report is recommended to complement but not substitute children's self-report |
| Vanmuilekom 2019 | Privacy ethical issues | Harder to discus | -Sometimes harder to discuss issues when parents/child are there |
| Lassen 2023 | Privacy ethical issues , Type of collection | Child sharing info, Parents helped completing self reports | -Some participants did not involve parents when filling out questionnaires and viewed this as an opportunity to share "secret" information with clinicians resulting in having a special bond with clinicians.  -Participants who had difficulty understanding questions explained that their parents helped them complete the questionnaire.  -These parents took part in pro-based dialogue with their children and clinicians indicating a partnership between parents, patient and clinicians. |
| Cox 2021 | Privacy ethical issues, Type of collection | Unclear whether to use child proxy, Both used | -Proxy measures problems: Providers must transition from proxy to self-report at 8 years old, then transition from PROMIS Paediatric to PROMIS adult measures.  -Requires decisions over which to use and how to interpret data. When both pt and proxy complete report, questions arise about how to use the information, especially when responses differ and when comparing scores over time. |
| Ryan 2013 | Type of collection | Both used | -Mixture of proxy and self. |
| Engelen 2010 | Type of collection | Proxy standard, Both used | -The PedsQL self-report form was used for children aged 8-12 and 13-18, while parents of children aged 6-7 completed the PedsQL proxy-report. The TAPQOL was added for parents of children aged 0-5 years. |
| Limperg 2013 | Type of collection | Proxy only for certain criteria, Both used | -Age 8-18 - self-report.  -Age under 7-proxy report |
| Salmond 2020 | Type of collection | Patient reported only | -All patient-reported |
| Taxter 2018 | Type of collection | Clinicians completed, Both used | -Completed by patient 39%, parent 48%, or clinic staff 14%. |
| Graham 2023 | Proxies and self-reports provide diff info | Discrepancies between reports | -Preference of PROMs depended on their condition and differences were also found between patients, caregivers and HCPs, |
| Robertson 2020 | Proxies and self-reports provide diff info | Proxies provide additional info, Discrepancies between reports | -Some discordance between CYP's answers and parents' opinions.  -Authors suggest giving parents opportunity to comment on PROMs independently.  -Parents have their own perspectives, some described feeling that they needed to correct their child's Reponses. |
| Blaauboer 2017 | Type of collection | Both used | -Both CYP and proxies completed PROMs. |
| Limperg 2012 | Type of collection | Both used,  Proxy only for certain criteria | -Age 8-18 self-report, under 8 proxy report. |
| Schepers 2016 | Type of collection | Both used | -Children aged 8-18 years self-reported  -Parents of patients aged 0-7 years reported |
| Fernandez-quintana 2021 | Proxies and self reports provide diff info | Proxies provide additional info | -Parental ratings of ASRS are more accurate than adolescent’s self-reports. |
| Townley 2019 | Type of collection | Both used | -Both used |
| Kroupina 2020 | Type of collection | Proxy standard | -ASQ (proxy) |
| Vanoers 2013 | Type of collection | Proxy only for certain criteria | -Proxies for younger children. |
| Franklin 2021 | Proxies and self reports provide diff info | Discrepancies between reports | -Significant difference between patient and their parent for certain domains. |
| Anthony 2021 | Proxies and self reports provide diff info | Proxies provide additional info | -Some participants valued proxy reports as they felt proxies might add information that patients might not share. |
| Mager 2019 | Type of collection | Both used | -Both collected. |
| Wheatbutt 2014 | Type of collection | Proxy standard, Both used | -Caregivers of children 17 and younger completed proxy assessments.  -Patients aged 11 and older completed self-report measures. |
| Haverman 2017 | Type of collection | Proxy only for certain criteria,  Both used | -Age 0-5 - TAPQOL – proxy.  -Age 6-7 - PedsQL – proxy.  -Age 8-18 - PedsQL - self-report.  -Only one informant used but clinicians trained to compare answers to child's own view. |
| Kuijlaars 2019 | Proxies and self reports provide diff info , Type of collection | Proxies provide additional info, Discrepancies between reports, Both used | -For children aged 4-7y, only parents completed PROM.  -Child and parent agreement varied across domains. The differences indicated that both child report and parent proxy should be reported. |
| Aldekhyyel 2018 | Type of collection | Both used | -Either could respond. |
| Wray 2019 | Type of collection | Proxy standard | -Parent reports only. |
| Vangorp 2021 | Type of collection | Proxy only for certain criteria, Both used | -2-7 years old patients use proxy PedsQL.  -8-18 years old used self-report. |
| Schepers 2013 | Type of collection | Both used | -Self report (age 8-18) or parents (child’s age 0-8). |
| Bjertnaes 2018 | Type of collection | Proxy standard | -Only parents surveyed |
| Haverman 2013 | Type of collection | Proxy only for certain criteria, Both used | -Patient-reports for ages 8-18 years old.  -Proxy-reports for aged 0-7 years old. |
| Maasalo 2017 | Proxies and self reports provide diff info | Discrepancies between reports.  Both used | -Some SDQ parents and CYPs agreed on reports whereas some disagreed. |
| Teela 2020 | Type of collection | Both used | -Both used. |
| Davis-dao 2020 | Type of collection | Proxy standard | -Surveys completed largely by caregivers. |
| Engelen 2012 | Type of collection | Proxy only for certain criteria, Both used | -Age 8-18y: PedsQL self-report.  -Age 6-7: PedsQL proxy-report.  -Age 0-5 TAPQOL proxy-report |
| Gmuca 2019 | Proxies and self reports provide diff info, Type of collection | Discrepancies between reports, Both used. | -Good to excellent agreement on all domains between pt and proxy.  -Older patient age associated with less agreement between patient self-report and parent-proxy report for psycho-social HRQoL |
| Knottnerus 2017 | Type of collection | Both used | -All questionnaires available to both. |
| Kemp 2018 | Type of collection | Proxy standard | -Proxy only |
| L'estrange-snowden 2018 | Type of collection | Proxy only for certain criteria, Both used | -Parent reports for children 0-7y.  -Self reports for 8-11y and 12-15y (space for carers to provide feedback). |
| Wolfe 2014 | Type of collection | Proxy standard | -2% of children aged 8y and over had parental reports.  -53% under 8 had parental reports |
| Wray 2017 | Type of collection | Proxy standard | -All proxy data |
| Wang 2018 | Type of collection | Proxy standard | -PROs only collected from parents. |
| Roesler 2018 | Type of collection | Proxy standard | -Only proxy reports |
| Haverman 2013 | Type of collection | Both used | -aged 8-18 years - self-report  -aged 0-18 years - proxy |
| Yao 2019 | Type of collection | Proxy standard | -Parent report only. |
| Wray 2020 | Type of collection | Proxy standard | -All parent-reported |
| Kermarrec 2015 | Type of collection | Proxy standard | -ASQ used (parent proxy) |
| Romo 2016 | Type of collection | Proxy standard | -All proxies |
| Murphy 2011 | Type of collection | Proxy standard | -All parent-reported |
| Haverman 2019 | Type of collection | Both used | -Children 8 years and above and parents/caregivers complete PROMs. |
| Riobueno-naylor 2019 | Type of collection | Proxy standard. | -BOQ and PSC both completed by parents.  - Child report version of BOQ available -but not used despite sample 5-18 yrs. |
| Uzark 2013 | Type of collection | Patient reported only | -Although PedsQL uses both, only child-report used in this study |
| Brann 2018 | Type of collection | Both used | -Patients and proxy both offered PROMs.  -Low completion of proxy PROMs for young adults and parents of young adults. |
| Limperg 2017 | Type of collection | Both used | -Children ages8-18 years self-report.  -Proxy report if they are < 8 years. |
| Tollit 2019 | Type of collection | Both used | -Mix of both |
| Katsicas 2011 | Type of collection | Both used | -Both used |
| Hall 2014 | Type of collection | Both used | -Both involved. |
